# Volume replacement in the resuscitation of trauma patients with acute hemorrhage: an umbrella review

**DOI:** 10.1186/s12245-023-00563-4

**Published:** 2023-11-30

**Authors:** Silvia Gianola, Greta Castellini, Annalisa Biffi, Gloria Porcu, Antonello Napoletano, Daniela Coclite, Daniela D’Angelo, Marco Di Nitto, Alice Josephine Fauci, Ornella Punzo, Primiano Iannone, Osvaldo Chiara, the Italian National Institute of Health guideline working group, Nino Stocchetti, Elvio De Blasio, Gaddo Flego, Massimo Geraci, Giulio Maccauro, Federico Santolini, Claudio Tacconi, Gregorio Tugnoli, Andrea Fabbri, Maria Pia Ruggieri, Carlo Coniglio

**Affiliations:** 1 Unit of Clinical Epidemiology, IRCCS Istituto Ortopedico Galeazzi, Milan, Italy; 2 Department of Statistics and Quantitative Methods, National Centre for Healthcare Research and Pharmacoepidemiology, University of Milano-Bicocca, Milan, Italy; 3 Department of Statistics and Quantitative Methods, Unit of Biostatistics, Epidemiology and Public Health, University of Milano-Bicocca, Milan, Italy; 4 Centro Nazionale Per L’Eccellenza Clinica, La Qualità E La Sicurezza Delle Cure, Istituto Superiore Di Sanità, Rome, Italy; 5 Azienda Sanitaria Locale Roma/6, Via Borgo Garibaldi, 12 00041 Albano Laziale, Rome, Italy; 6 CECRI Evidence-Based Practice Group for Nursing Scholarship: A JBI Affiliated Group, Rome, Italy; 7 Dipartimento Di Medicina Interna, Azienda USL, Ospedale Maggiore, Largo Nigrisoli 2, 40133 Bologna, Italy; 8 Department of Pathophysiology and Transplantation, University of Milan, Milan, Italy; 9 General Surgery and Trauma Team, ASST Grande Ospedale Metropolitano Niguarda, University of Milan, Milano, Piazza Ospedale Maggiore, Milan, Italy

**Keywords:** Systematic review, GRADE approach, Major trauma, Emergency treatment, Fluid therapy

## Abstract

**Background:**

The use of intravenous fluid therapy in patients with major trauma in prehospital settings is still controversial. We conducted an umbrella review to evaluate which is the best volume expansion in the resuscitation of a hemorrhagic shock to support the development of major trauma guideline recommendations.

**Methods:**

We searched PubMed, Embase, and CENTRAL up to September 2022 for systematic reviews (SRs) investigating the use of volume expansion fluid on mortality and/or survival. Quality assessment was performed using AMSTAR 2 and the Certainty of the evidence was assessed with the Grading of Recommendations Assessment, Development, and Evaluation (GRADE) approach.

**Results:**

We included 14 SRs investigating the effects on mortality with the comparisons: use of crystalloids, blood components, and whole blood. Most SRs were judged as critically low with slight overlapping of primary studies and high consistency of results. For crystalloids, inconsistent evidence of effectiveness in 28- to 30-day survival (primary endpoint) was found for the hypertonic saline/dextran group compared with isotonic fluid solutions with moderate certainty of evidence. Pre-hospital blood component infusion seems to reduce mortality, however, as the certainty of evidence ranges from very low to moderate, we are unable to provide evidence to support or reject its use. The blood component ratio was in favor of higher ratios among all comparisons considered with moderate to very low certainty of evidence. Results about the effects of whole blood are very uncertain due to limited and heterogeneous interventions in studies included in SRs.

**Conclusion:**

Hypertonic crystalloid use did not result in superior 28- to 30-day survival. Increasing evidence supports the scientific rationale for early use of high-ratio blood components, but their use requires careful consideration. Preliminary evidence is very uncertain about the effects of whole blood and further high-quality studies are required.

**Supplementary Information:**

The online version contains supplementary material available at 10.1186/s12245-023-00563-4.

## Introduction

Traumatic injury is one of the leading causes of death worldwide, representing approximately 10% of global deaths, especially in the young population [1]. Uncontrolled bleeding is responsible for approximately 50% of deaths within 24 h after the injury [2, 3] and represents the main cause of potentially preventable death in trauma patients. Uncontrolled bleeding requires quick identification and immediate action to save the patient [4]. The early recognition of hemorrhages in pre-hospital settings reduces the activation time of hospital massive transfusion protocols [5], counteracts the progression of acute traumatic-induced coagulopathy [6, 7] and is associated with improved survival in severely injured trauma patients [8]. Early treatment in the prehospital setting, and emergency department (ED) included intravenous fluid administration and/or blood transfusion [9, 10] to increase intravascular volume, cardiac preload and output, global oxygenation, and microvascular perfusion [11].

Judicious fluid resuscitation is the first step in the hemodynamic management of patients with hemorrhagic shock to avoid an increase in bleeding and protect the patient from severe consequences of hypovolemic shock [12]. A multidisciplinary Task Force for Advanced Bleeding Care of the European guideline on the management of major trauma recommends a prehospital assessment of the circulation status [13] with the resuscitation of patients with traumatic hemorrhagic shock aimed at maintaining a systolic blood pressure of 80–90 mmHg, limited infusion of crystalloids/colloids [14] and increased early blood component transfusion until definitive bleeding control [8]. Since then, several systematic reviews have been performed to analyze which fluids should be used for patients with major trauma in hemorrhagic shock. However, there is still no consensus on the optimal resuscitation strategy with respect to the type, quantity, and timing of fluid therapy [15].

Volume replacement is still cornerstones to correct fluid deficits during early trauma resuscitation, but the conduct and reporting of literature are various in terms of purpose and outcome measurements. The aim of this umbrella review is to map the scientific literature on the type of resuscitation fluids and establish an overall view of the available evidence on their use in trauma patients with hemorrhagic shock.

## Methods

We applied the Cochrane method for overviews of reviews and the JBI guidelines for umbrella reviews to systematically map the use of expansion fluids in the resuscitation of trauma patients with acute hemorrhage to reduce mortality [16, 17]. The study protocol has been stored at the following link: https://osf.io/86k5u/. The report has been written according to the Preferred Reporting Items for Overviews of Reviews (PRIOR) [18].

### Clinical question and study design

We conducted an overview of reviews to support the major trauma integrated management guideline panel of our Institute of Health [19, 20] in formulating recommendations for the following clinical question: *What is the best volume expansion fluid to use in the resuscitation of hemorrhagic shock?* We used the Grading of Recommendations Assessment, Development and Evaluation (GRADE)-ADOLOPMENT approach for guideline production [21], adopted by the national methodological manual [22], to guide a structured and systematic adaptation and updating process of the recommendation on the volume expansion fluid to use in the resuscitation of hemorrhagic shock from NICE NG39 [23].

### Inclusion/exclusion criteria

We included studies if they were systematic reviews of original research (randomized controlled trials and observational studies), with or without meta-analyses that investigated the effectiveness of different kinds of fluid replacement in patients with hemorrhagic shock after a traumatic incident. We defined a systematic review, based on Cochrane’s definition, as a review of the literature in which one “attempts to identify, appraise and synthesize all the empirical evidence that meets pre-specified eligibility criteria to answer a specific research question by using explicit, systematic methods that are selected with a view aimed at minimizing bias, to produce more reliable findings to inform decision making” [24]. We excluded narrative reviews, non-systematic literature reviews, and systematic reviews of material that was not original research (e.g., systematic reviews of guidelines). Eligibility criteria for the overview were established using the Population, Intervention, Comparator, Outcome framework to include: (1) *Population*: adults who have experienced a traumatic incident. Pre-hospital and emergency department (ED) have been considered because initial management in ED is a prolongation of the pre-hospital treatment (*continuum of care* concept); (2) *Interventions*: crystalloids, packed red blood cells (PRBCs), fresh frozen plasma (FFP), platelets (PLT), liquid plasma, lyophilized plasma, low titer 0-negative whole blood (LT0WB); (3) *Comparisons*: a comparison or combination of the above (including different ratios). We defined *blood component therapy* as “plasma and/or PRBCs (with or without crystalloids)” whereas we defined *standard care* as “infusion with crystalloids, no transfusion, unknown or combination with blood components” [25]; (4) *Outcome*: overall mortality and/or survival (e.g., 24 h, 30 days/1 month). We excluded people with a major trauma resulting from burns, and patients in hypovolemic shock not caused by trauma.

### Search strategy

We used the search strategy performed by the adopted NICE guideline [23] from 26, March 2015 up to 9 September 2022 the following electronic databases: MEDLINE (PubMed), EMBASE (Elsevier, EMBASE.com), and Cochrane Central Register of Controlled Trials (CENTRAL) restricted to English only. This time span has been chosen as this overview represents an update of the systematic review carried out by the NICE to develop recommendations for the NG39 [23]. Searching for the research synthesis conducted within the last 5 to 10 years yields primary research covered at least 20 years prior [17].

The search strategy is outlined in Additional file 1: Appendix 1, Supplement 1. The literature search was supplemented by reviewing the bibliographies of the included reviews and other key papers.

### Selection of studies and data extraction

Two independent authors (SG, GC) screened titles and abstracts obtained with the selected search strategy. Each reviewer then independently assessed the full text of potentially relevant studies for inclusion. Any disagreement was solved by a discussion with a third reviewer (OC). We adopted a standardized data collection form to extract the following information: review characteristics (e.g., year of conduct/literature search, type of included study designs), patient characteristics (e.g., type and number of patients, age mean), and interventions examined (e.g., type of intervention, ratio of intervention). A list of the primary studies included in all the systematic reviews with meta-analysis was compiled and cross-referenced with the primary studies included in the SRs. We contacted authors if the reported data were insufficient or unclear.

### Quality appraisal and certainty of the evidence

Quality appraisal was completed concurrently with data extraction using the Assessment of Multiple Systematic Reviews 2 (AMSTAR 2) Checklist tool [26], an updated version of the original AMSTAR [27], specifically developed to assess the methodological quality of systematic reviews of randomized and non-randomized studies of healthcare interventions. Two independent authors (MD, DD) performed the assessment, and any disagreement was solved by consensus. We carried out descriptive tables of the quality of the primary studies reporting the rating shown in the included SRs. Additionally, the Certainty of the Evidence (CoE) was assessed by two authors (AB, GP) using the GRADE approach examining five dimensions (risk of bias, consistency of effect, imprecision, indirectness, and publication bias) [28]. The evidence was downgraded from “high quality” by one level or by two levels if, respectively, serious or very serious limitations were found for each of the five dimensions. Reasons for limitation and main findings are reported in a tabular format presenting the “summary of findings” of the certainty of the evidence for the mortality outcome (e.g., 24 h, 30 days/1 month).

### Data synthesis

We adhered to all data collection and synthesis methodology outlined in the Cochrane Handbook’s chapter on overviews of reviews and JBI Manual for Evidence Synthesis [17, 29]. No formal statistical analysis was planned for this overview as substantial clinical and methodological heterogeneity was expected across the included reviews and pooling the data or conducting an indirect comparison would not be appropriate in this situation.

We summarized the main results of the included reviews by categorizing their findings in the following comparisons (1) use of crystalloids, (2) use of blood components (a) and their blood components ratios (b), (3) use of whole blood (WB). Lists of the primary studies in each included review were collated and cross-referenced in the matrix of evidence tables to ascertain the degree of overlap between reviews for each treatment comparison of mortality outcome and to provide context for the results. Additionally, a matrix of evidence for the entire overview was prepared and used to calculate the “corrected covered area” (CCA) to quantify the degree of overlap between all the reviews included in this work [30].

## Results

### Study selection

**Fig. 1:**
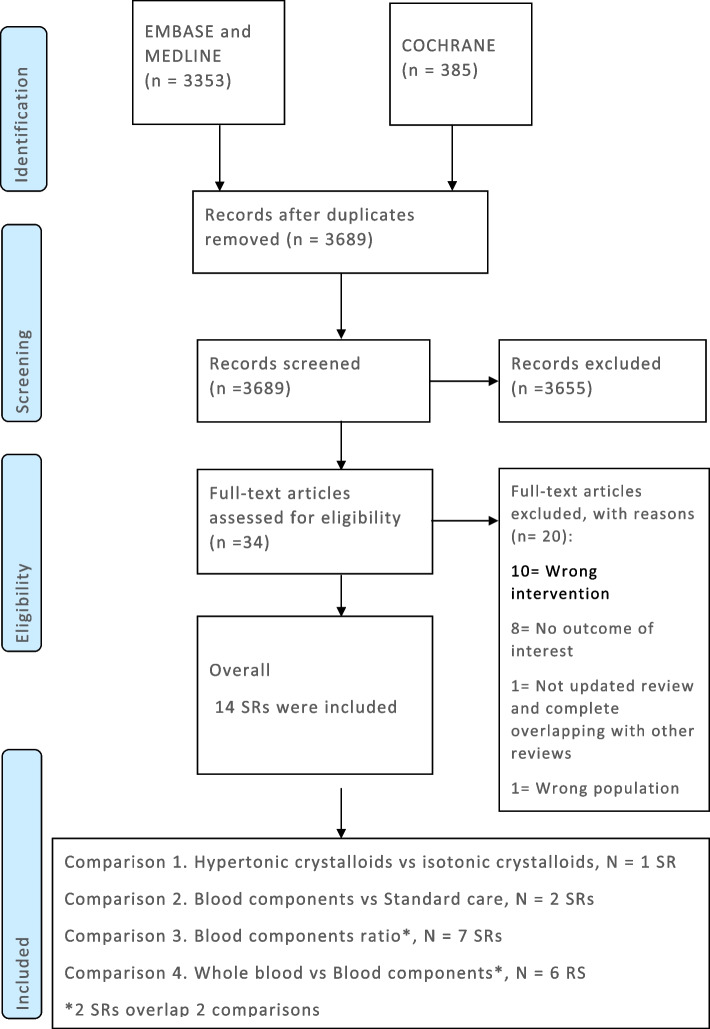
Study flow diagram

A total of 3689 records were screened, 3655 were excluded after the title and abstract screening while 20 were excluded after a full-text reading (Additional file 1: Appendix 1, Supplement 2). Overall, 14 systematic reviews were included, the study flow diagram is reported in Fig. 1.

### Review characteristics

The included reviews were conducted between 2018 and 2021 with half (50%) published in 2020. Literature search dates for the included reviews ranged from 2015 to 2022 (min–max). A total of 210 studies were included in the SRs with 46 randomized controlled trials and 164 observational studies. Most SRs (79%) performed a meta-analysis. Characteristics of included studies are reported in Table 1 and further details are presented in Additional file 1: Appendix 1, Table S1.

**Table 1:** Summary of general characteristics of included systematic reviews

Characteristics	Systematic reviews
Year of publication [median (range)]	2020 (2019–2020)
Literature search date [median (range)]	2019 (2018–2020)
Included study designs [median (range)]	8 (5–14)
Randomized controlled trials [median (range)]	2 (1–5)
Observational studies [median (range)]	6 (0–11)
Overall sample size [median (range)]	3260 (1408–7603)
Meta-analysis [*N* (%)]	11(79)

### Overlap between included SRs and studies

In Table 2, we mapped all SRs covering the three included comparisons. Between the 14 SRs included, two [31, 32] overlapped between the comparisons “blood component ratio” and “whole blood vs blood component therapy”. A total of 86 primary studies were cited 136 times across the 14 SRs included in this overview, resulting in a CCA of 0.04 indicating little to no overlap across the included reviews meaning that SRs addressed different clinical intervention questions (Additional file 1: Appendix 1, Table S2–S4).

**Table 2:**
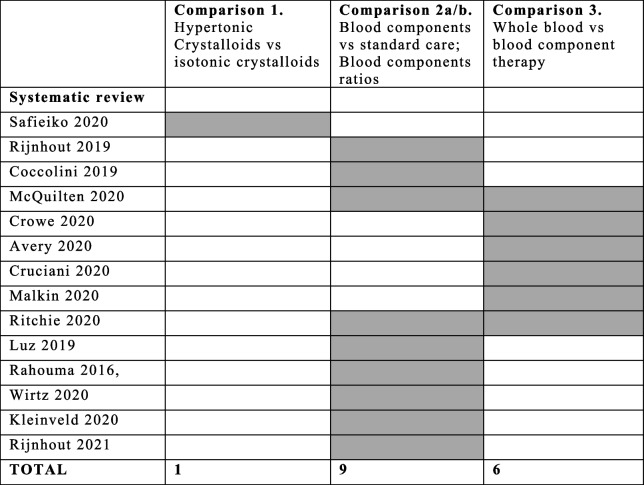
Map of included SRs across comparisons

*Legend*: In grey the included SRs per each comparison

### Comparison 1: hypertonic crystalloids vs isotonic crystalloids

We found one systematic review of RCTs investigating hypertonic saline/dextran or hypertonic saline versus isotonic fluid [33]. This systematic review does not present the superiority of the hypertonic saline/dextran group compared with isotonic fluid solutions at 28- to 30 days survival (primary endpoint) (5 RTCs *n* = 1440, OR = 1.13; 95% CI 0.75–1.70; *I* ^2^ = 43%; *p* = 0.56). Nevertheless, the results were statistically significant for the secondary endpoints, 24 h survival (4 RCTs, *n* = 807, OR = 2.99; 95% CI 2.04–4.39; *I* ^2^ = 0%; *p* < 0.001), and overall mortality (7 RCTs, *n* = 1962, OR = 0.76; 95% CI 0.61–0.94; *I* ^2 ^= 33%; *p* = 0.01) respectively. Additional file 1: Appendix 1, Table S3 shows the outcome assessed in detail.

### Comparison 2: blood components

#### 2a. Use of blood components vs standard care

We found two SRs addressing this comparison in the pre-hospital setting. Specifically, one systematic review included observational studies and RCTs investigating the effect of both PRBCs alone and plus plasma versus standard care [25] and the other one systematic review included only RCTs assessing plasma versus standard care [25, 34]. Additional file 1: Appendix 1, Table S4 reports all the outcome data on mortality. PRBCs plus plasma resulted in a reduction in the odds of mortality at 30 days (1 RCT: OR = 0.51, 95% CI, 0.33–0.81, *p* < 0.0001; 3 observational studies: OR = 0.49; 95% CI, 0.29–0.83, *p* = 0.008), while no superiority was found between PRBCs alone when compared to standard care (4 observational studies: OR = 1.18; 95% CI, 0.93–1.49, *P* = 0.17). Pre-hospital plasma infusion seems to reduce 24-h mortality in haemorrhagic shock patients (2 RCTs: RR = 0.69; 95% CI = 0.48–0.99).

#### 2b. Blood components ratios

We found different ratios of blood components use: (I) FFP: PLT: PRBC, (II) FFP: PRBC, (III) PLT: PRBC in which we found 3 systematic reviews [31, 32, 35], 2 systematic reviews [35, 36], and 4 systematic reviews [24, 35, 37, 38] respectively. In general, a high ratio was superior compared to a low ratio (FFP: PRBC; PLT: PRBC). Additional file 1: Appendix 1, Table S5 reports all the outcome data on mortality.

### Comparison 3: whole blood components vs component therapy

Six systematic reviews investigated the comparison of WB components vs component therapy (e.g., PRBC + plasma) [31, 32, 39–43]. All SRs included the same unique RCT [44] and some observational studies conducted in the ED setting. With complete agreement among reviews, no superiority of WB versus any blood component therapy was found. Additional file 1: Appendix 1, Table S6 reported mortality data of the study designs included.

### Quality appraisal and assessment of the evidence

**Fig. 2:**
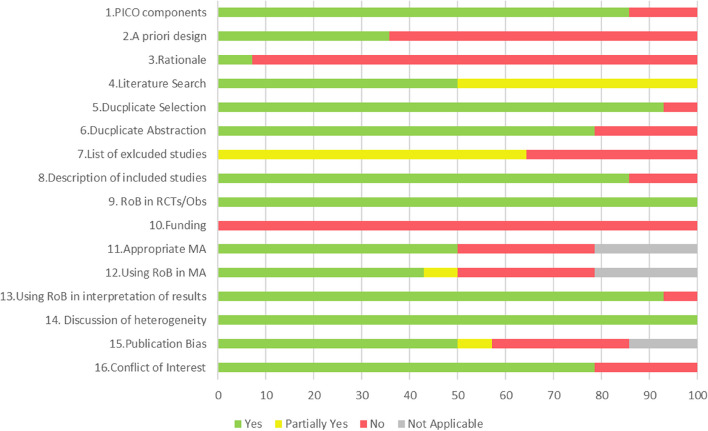
Quality appraisals across included SRs (AMSTAR 2)

The quality appraisal of included SRs ranged from critically low quality to moderate quality and overall items are reported in Fig. 2. Most SRs are judged critically low (64%), followed by those with low (29%) and moderate (7%) ratings.

Consistency of evidence was found in all comparisons. The certainty of the evidence was heterogeneous across comparisons and outcomes and ranged from very low to high according to comparisons and outcomes. Table 3 reports a summary of the certainty of the evidence whereas the reasons for downgrading and more details are reported in Additional file 1: Appendix 1, Supplement 3.

**Table 3:** Summary of certainty of evidence across mortality outcomes from SRs

Comparisons	SR	24 h mortality	30 days mortality	Overall mortality
1: Hypertonic crystalloids vs isotonic crystalloids 2. Blood components	1	High	Moderate	high
2a: Blood products versus standard care				
PRBCs versus standard care	1		Very low	Very low
PRBCs and plasma versus standard care	1	Very low	Moderate (RCT); low (obs)	
Plasma versus standard care	1	Low	Low	
		Moderate	Low	
2b: Blood components ratios				
FFP:PLT:PRBC ratio	3	Very low	Very low	
		Low	Very low	
		Very low	Very low	
FFP:PRBC ratio, 1:1 vs < 1:1	2	Very low	Very low	
		Low	Low	
FFP:PRBC ratio, 1:1.5 vs < 1:1.5	2	Very low	Very low	
		Very low	Very low	
FFP:PRBC ratio, 1:2 vs < 1:2	2	Low	Very low	
		Low	Low	
PLT:PRBC ratio, 3.3.1 ≥ 1:1 vs < 1:1	1	Very low		
PLT:PRBC ratio, ≥ 1:9 vs < 1:9	1	Very low		
PLT:PRBC ratio, > 1:20 vs 1:1	1	Very low	Very low	
PLT:PRBC ratio, 1:2 vs 1:1	1	Very low	Low	
PLT:PRBC ratio, 1:8 vs 1:16	1	Very low	Very low	
PLT:PRBC ratio, ≥ 1:2 vs < 1:2	1	Very low		
PLT:PRBC ratio, ≥ 0.1 vs ≤ 0.1	1	Very low	Very low	
PLT:PRBC ratio, ≥ 0.3 vs < 0.3	1	very low		
PLT:PRBC ratio, ≥ 0.5 vs < 0.5	1	Very low	Very low	
PLT:PRBC ratio, ≥ 1 vs 0.5 or < 1	1	Very low		
PLT:PRBC ratio, ≥ 1 vs 0.6 or < 1	1		Very low	
PLT:PRBC ratio, high vs low	1	Moderate	Moderate	
3: Whole blood components vs blood component therapy	3	Very low	Very low	
		Very low	Very low	
		Very low	Very low	

*Legend*: *PRBCs* packed red blood cells, *FFP* fresh frozen plasma, *PLT* platelets, *obs* observational study, *RCT* randomized controlled trial

## Discussion

This umbrella review explores the evidence which has been published about initial resuscitation from hemorrhagic shock in trauma patients. The setting of intervention considered was the pre-hospital and emergency department care.

With respect to the crystalloids comparison, the use of hypertonic saline/dextran compared to isotonic fluid solutions did not result in a superiority at the primary endpoint 28- to 30-day survival (moderate to high CoE), while an improvement of the survival at 24 h, and less overall mortality (secondary endpoints) in patients with hypotension were found. The results of 28- to 30-day survival have been confirmed by a recent systematic review with meta-analysis in patients with severe traumatic brain injury that has shown no better survival for patients treated with hypertonic saline when compared to normotonic crystalloids (overall RR 0.99, 95% CI 0.93–1.06) [45]. Furthermore, hypertonic saline infusion has been associated with an increase in uncontrolled hemorrhage and coagulation disorders in dextran-containing solutions [46], and cases of pontine osmotic demyelination have been described with the normalization of osmolarity after hypertonic infusion [47, 48]. This latter condition has been reported in patients with hyponatremic states when sodium levels have been corrected rapidly. Its incidence is not well-known due to under-diagnosis, but retrospective study from 2015 shows the incidence of osmotic demyelination syndrome is 2.5% among intensive care unit (ICU) admissions [49]. Based on these clinical considerations and considering the unclear information on how to use intravenous hypertonic (concentration, formulation, or volume) comprehensive recommendations on hypertonic saline are difficult to draw.

Within the blood components comparison, the PRBCs compared to standard of care have shown little to no effect on mortality at 24 h as well as at 30 days with very uncertain (very low CoE) level of evidence. Adding plasma to PRBCs has shown to be effective in reducing 30-day mortality (low to moderate CoE), and using plasma alone seems to reduce the 24-h mortality compared to standard care (moderate CoE). These results are consistent with a secondary analysis from a non-randomized study which reported that the use of fresh frozen plasma in combination with packed red cells was beneficial, compared to usual care [50]. Conversely, a very recent and large RCT [51] on the use of PRBC and lyophilized plasma versus sodium chloride in adult patients with trauma-related hemorrhagic shock has shown a non-superiority of PRBC–LyoPlas resuscitation in reducing mortality. Based on our results in conjunction with the most recent studies, no hard conclusion can be drawn about a possible benefit for hemorrhagic trauma patients receiving blood components. Nevertheless, it is important to highlight that the established trends in damage control resuscitation about the “hemostatic resuscitation” suggest an early use of blood products rather than an abundance of crystalloids in order to minimize tissue edema with metabolic derangement and organ failure, and hemodilution with resuscitation-induced coagulopathy [39]. Indeed, in the current 10th edition of the ATLS guidelines [52] only 1 L of crystalloid (including the amount in the pre-hospital setting) is suggested. Moreover, in trauma patients with uncontrolled bleeding foci, volume infusion should be limited to the lowest acceptable pressure value, to prevent re-bleeding from the injured vascular bed [13]. However, the administration of blood products compared to crystalloids may avoid a dilution of coagulation factors, leading to a higher perfusing pressure since blood will maintain hemostasis, and prevent the deleterious effects of primary and secondary coagulopathy [53]. Considering the above, the generalizability of the results of this comparison can be challenging in several ways, mainly because several mechanisms could explain the effect of these interventions on mortality, such as reduction in bleeding or coagulopathy, a diminution of the inflammatory response, or endothelial dysfunction of trauma. More studies are needed to identify specific patient cohorts that may benefit from blood components transfusion and explore the effects of different transfusion strategies, also considering transportation times (short/long) from the scene to the emergency department.

Our overview identifies seven SRs comparing different ratios of blood components used (FFP: PLT: PRBC; FFP: PRBC; PLT: PRBC). With complete concordance among all SRs, we found significant results in favor of higher ratios among all comparisons considered (Additional file 1: Appendix 1, Supplement 3). Regarding FFP:RBC ratio, the range considered was between 1:1 and 1:2, confirming that higher ratios were beneficial in terms of 24 h and 30 days of mortality after the traumatic event. This result is in line with the guidelines published by the American College of Surgeons [54], advocating an FFP:RBC ratio between 1:1 and 1:2 and other published studies [55–57], although all of the cited documents are not specific to the prehospital setting.

Regarding WB use compared to component therapy, all SRs agreed to no difference between interventions in terms of reduced mortality for patients with major trauma. The use of this intervention can be contextualized on studies conducted in the ED setting considering that our SRs included different types of WB (e.g., low titer cold stored O-negative WB (LTOWB), fresh WB, unrefrigerated young WB, and unspecified WB). Recent developments show promising effects during the prehospital or in-hospital setting. A recent pilot RCT, showed no statistical mortality benefit at 28 days when compared to standard care (25.0% vs. 26.1%, *p* = 0.85) even though patients randomized to prehospital LTOWB had lower red cell transfusion requirements at 24 h (*p* < 0.01), lower incidence of Multiple Organ Failure and no transfusion reactions [58]. In addition, from recent observational studies, whole blood transfusion compared to blood components emerged with faster resolution of shock, lower post-transfusion INR, decreased component product transfusion^43^, improved survival, and decreased overall blood utilization [59, 60], with no increased risk of adverse events (e.g., MODS) [43, 60].

Pros and Cons of WB should be acknowledged. WB use could avoid the loss of quality of blood product components, as WB has a longer storage time. Indeed, the WB has a longer shelf life (14–35 days) compared to room temperature stored platelets (5 days), and WB increases the storage duration of platelets 3 to 7 times more than a platelet concentrate [61]. The use of WB can also limit the number of infusions received from different donors, thus reducing the risk of blood-borne pathogens. Additionally, the transfusion of a single WB unit (especially LTOWB) is simpler than transfusing multiple components and may reduce harm from administrative errors [15]. It is also interesting to note how recent efforts have been focused on the use of low-titer 0 negative WB (LTOWB), with clear advantages for recipients compared to other blood types (e.g., faster resolution of shock, lower post-transfusion INR, and decreased component product transfusion) (Leeper REF). However, the procurement of donors with low titer 0 negative of IgM/IgG antibodies Anti-A and Anti-B is not easy [62].

## Limitations

However, some limitations should be underlined. The reporting and conduct of our sample were heterogeneous in terms of purpose and outcome measurement. In fact, we included SRs that addressed different clinical intervention questions (CCA indicating little to no overlap across reviews), and around two-thirds were of critically low quality. As a note of this clinical heterogeneity, we included SRs focussed on different trauma scenarios. For example, some evidence comes from pre-hospital (e.g., comparison 2 on blood component) or ED setting (e.g., comparison 4 on whole blood) as a continuum of care. The clinical heterogeneity also regards the multiple confounding factors. For example, in the blood components comparison, the two SRs differently classify the intervention of the PAMPER trial [10] (plasma in Coccolini et al.2019 [25, 34], plasma + pRBCs in Rijnhout et al. 2019 [25, 34]) as the primary authors declared that they cannot determine the independent or additive effects of prehospital administration of plasma and packed red cells. Analogously, in the whole blood comparison the unique RCT [44], reported in all SRs, declared the use of crystalloids and colloids during resuscitation.

## Implication for practice and future research

Crystalloids represent a way to obtain a volume expansion in hemorrhagic trauma patients on the scene when blood products are not available, or it may be difficult to administer other forms of intravenous fluid therapy to restore intravascular volume [33]. However, crystalloid infusion is associated with tissue edema, organ failure, and dilutional coagulopathy [15] and their use should be carefully evaluated based on the specific clinical case.

Our results support the infusion of blood components with high FFP and PLT ratios [24, 32, 36, 38], as soon as possible in the process of care of trauma patients, from the pre-hospital care to the emergency department (ED). However, based on mainly poor-quality evidence no hard conclusion can be drawn about a possible survival benefit for hemorrhagic trauma patients receiving a pre-hospital blood-component transfusion. Once in ED, the transfusion of blood components with a high FFP/PLT/PRBC ratio should be the primary way for volume replacement until definitive control of bleeding foci, as suggested by the overlapping of three SRs.

To support the major trauma-integrated management guideline of our Institute of Health the panel provided the following recommendation. Using blood components in the earliest phase (pre-hospital setting including ED) is suggested in patients with hemorrhagic shock and coagulopathy. (weak recommendation based on low certainty of evidence) (LGTM-consultazione-Racc23-24_25_report_def (iss.it)).

Indeed, based on the most recent literature and the physiopathology rationale [15], the implementation of blood components transfusion in the pre-hospital setting could be a goal for healthcare organizations. This means a more efficient involvement of blood transfusion services in the trauma center network, with an organization allowing the daily availability of blood or blood components on ambulances or helicopters.

Future efforts should be devoted to improving the organization, skills, and competence of the personnel dedicated to blood transfusion. Pre-hospital care should enhance the adoption of standards and quality indicators at the international level [63]. This includes the involvement of blood transfusion centers in the planning and development of protocols with special attention to conservation, transportation, and utilization of products as well as for prevention and notification of adverse events.

## Conclusion

Based on the results of this overview, clinicians should consider volume expansion in resuscitation as a first-line intervention. Hypertonic crystalloid use did not result in a reduction of 28- to 30-day survival, supported by moderate certainty of evidence. Increasing evidence supports the scientific rationale for the early use of blood components with a high ratio to reduce mortality even though their use requires careful considerations, and no firm conclusion can be drawn. Preliminary evidence is very uncertain about the effects of WB due to limited and heterogeneous interventions in studies included in SRs. Further efforts should be made in conducting high-quality RCTs needed to draw more accurate conclusions on blood components or WB transfusion.

### Supplementary Information

**Additional file 1: Appendix 1: Supplement 1.** Search strategy and PICO question. **Table S1.** Detailed general characteristics of included systematic reviews. **Table S2.** Map of primary studies contained within each included systematic review per different comparison. **Table S3.** Mortality/survival, comparison hypertonic crystalloids vs isotonic crystalloids. **Table S4.** Mortality, use of blood components. **Table S5.** Mortality, blood components ratios. **Table S6.** Mortality, whole blood components. **Supplement 2.** Characteristics of excluded SRs. **Supplement 3.** Summary of findings.

## Data Availability

All data generated or analyzed during this study are included in this published article (and its additional files, https://osf.io/86k5u/).
