# Supplementary material for: Volume replacement in the resuscitation of trauma patients with acute hemorrhage: an umbrella review
Source: Int J Emerg Med. 2023 Nov 30;16:87. doi: 10.1186/s12245-023-00563-4 (PMC10687916; doi:10.1186/s12245-023-00563-4)
Supplement: Supplementary file 1 — Additional file 1: Appendix 1: Supplement 1. Search strategy and PICO question. Table S1. Detailed general characteristics of included systematic reviews. Table S2. Map of primary studies contained within each included systematic review per different comparison. Table S3. Mortality/survival, comparison hypertonic crystalloids vs isotonic crystalloids. Table S4. Mortality, use of blood components. Table S5. Mortality, blood components ratios. Table S6. Mortality, whole blood components. Supplement 2. Characteristics of excluded SRs. Supplement 3. Summary of findings. [file 12245_2023_563_MOESM1_ESM.docx]

Appendix 1

# Supplement 1. Search strategy and PICO question

| **Review question: What is the best volume expansion fluid to use in the resuscitation of hemorrhagic shock?** | |
| --- | --- |
| Objective: To determine what type of fluid replacement should be used in the management of shock for children, young people and adults who have experienced a traumatic incident. | |
| Population | Children, young people and adults who have experienced a traumatic incident. |
| Intervention | Red blood cells  Fresh frozen plasma  Liquid plasma  Crystalloids  Lyophilized plasma  Low Titer 0-negative Whole Blood |
| Comparison | A comparison or combination of the above (including different ratios) |
| Outcomes | Critical:  Mortality at 24 hours, 30 days/1month and 12 months  Health related quality of life  Length of intensive care stay  Adverse effects: (check SHOT website for acute transfusion reactions)  Acute transfusion reaction:  Hemolytic transfusion reaction – acute  Hemolytic transfusion reaction – delayed  Post transfusion purpura  Previously uncategorized complications of transfusion  Transfusion associated graft versus host disease  Transfusion associated circulatory overload  Transfusion associated dyspnea  Transfusion related acute lung injury  Transfusion transmitted infections    Important:  Time to definitive control of hemorrhage Patient-reported outcomes: return to normal activities psychological wellbeing) |
| Exclusion | People with a major trauma resulting from burns. Patients in shock, not from trauma. |
| Search strategy | Databases: Medline, Embase, the Cochrane Library  Date: All years  Language: English, French, Spanish, German, Italian  Study designs: RCTs or Systematic reviews of RCTs; cohorts |

**Standard major trauma POPULATION**

**Medline search terms**

| 1. | (trauma* or polytrauma*).ti,ab. |
| --- | --- |
| 2. | ((serious* or severe* or major or life threaten*) adj3 (accident* or injur* or fall*)).ti,ab. |
| 3. | multiple trauma/ |
| 4. | wounds, gunshot/ or wounds, stab/ or accidents, traffic/ or accidental falls/ or blast injuries/ or accidents, aviation/ |
| 5. | ((motor* or motorbike* or vehicle* or road or traffic or car or cars or cycling or bicycle* or automobile* or bike* or head on or pile up) adj3 (accident* or crash* or collision* or smash*)).ti,ab. |
| 6. | (mvas or mva or rtas or rta).ti,ab. |
| 7. | (stabbed or stabbing or stab or gunshot* or gun or gunfire or firearm* or bullet* or knife* or knives or dagger).ti,ab. |
| 8. | or/1-7 |

**Embase search terms**

| 1. | (trauma* or polytrauma*).ti,ab. |
| --- | --- |
| 2. | ((serious* or severe* or major or life threaten*) adj3 (accident* or injur* or fall*)).ti,ab. |
| 3. | multiple trauma/ |
| 4. | gunshot injury/ or stab wound/ or traffic accident/ or falling/ or blast injury/ or aircraft accident/ |
| 5. | ((motor* or motorbike* or vehicle* or road or traffic or car or cars or cycling or bicycle* or automobile* or bike* or head on or pile up) adj3 (accident* or crash* or collision* or smash*)).ti,ab. |
| 6. | (mvas or mva or rtas or rta).ti,ab. |
| 7. | (stabbed or stabbing or stab or gunshot* or gun or gunfire or firearm* or bullet* or knife* or knives or dagger).ti,ab. |
| 8. | or/1-7 |

**Cochrane search terms**

| #1. | MeSH descriptor: [multiple trauma] this term only |
| --- | --- |
| #2. | (trauma* or polytrauma*):ti,ab |
| #3. | ((serious* or severe* or major) near/3 (accident* or injur* or fall*)):ti,ab |
| #4. | MeSH descriptor: [wounds, gunshot] this term only |
| #5. | MeSH descriptor: [wounds, stab] this term only |
| #6. | MeSH descriptor: [accidents, traffic] this term only |
| #7. | MeSH descriptor: [accidental falls] this term only |
| #8. | MeSH descriptor: [blast injuries] this term only |
| #9. | MeSH descriptor: [accidents, aviation] this term only |
| #10. | ((motor* or motorbike* or vehicle* or road or traffic or car or cars or cycling or bicycle* or automobile* or bike*) near/3 (accident* or crash* or collision* or smash*)):ti,ab |
| #11. | (mvas or mva or rtas or rta):ti,ab |
| #12. | (stabbed or stabbing or stab or gunshot or gun or gunfire or firearm* or bullet or knife* or knives or dagger or shot):ti,ab |
| #13. | {or #1-#12} |

**INTERVENTION**

**Medline search terms**

| 1. | *standard trauma population (see F.2.1)* |
| --- | --- |
| 2. | hemorrhage/ or exsanguination/ or shock/ or shock, hemorrhagic/ or shock, traumatic/ or hypovolemia/ |
| 3. | (hypovol?em* or shock or exsanguin* or olig?em* or h?emorrhag* or hypoperfus*).ti,ab. |
| 4. | (coagulopath* or (abnormal* adj2 coagulation) or hyperfibrinolysis).ti,ab. |
| 5. | (bleed* or bloodloss*).ti,ab. |
| 6. | (blood* adj3 loss*).ti,ab. |
| 7. | or/2-6 |
| 8. | ((red blood cell* or rbc or prbc or red cell* or blood or packed cell* or erythrocyte*or fluid* or volum* or plasma*) adj5 (therap* or transfus* or replac* or resuscita* or substitut* or restor* or deficien* or replenish*)).ti,ab. |
| 9. | exp plasma/ |
| 10. | (ffp or ((frozen or thawed or tp or fresh) adj3 plasma)).ti,ab. |
| 11. | (albumin or zenalb or octaplas*).ti,ab. |
| 12. | ((lyophili?ed or freeze-dried or liquid or "not frozen" or "never frozen") adj3 plasma).ti,ab. |
| 13. | (fdsp or fdp or lqp or lhp).ti,ab. |
| 14. | exp freeze drying/ and plasma.ti,ab,sh. |
| 15. | exp sodium chloride/ |
| 16. | exp fluid therapy/ |
| 17. | exp rehydration solutions/ |
| 18. | exp plasma substitutes/ |
| 19. | exp isotonic solutions/ |
| 20. | (sodium or salin* or hartman* or ringer* or lactate* or acetate* or plasmalyte* or plasmalyte*).ti,ab. |
| 21. | (crystalloid* or isotonic).ti,ab. |
| 22. | ((balanced or physiologic*) adj2 (fluid* or solution*)).ti,ab. |
| 23. | or/8-22 |
| 24. | 1 and 7 and 23 |
| 25. | *blood transfusion/ or exp *blood component transfusion/ or *exchange transfusion, whole blood/ or *plasma exchange/ |
| 26. | 24 or 25 |

**Embase search terms**

| 1. | *standard trauma population (see F.2.1)* |
| --- | --- |
| 2. | exp *hypovolemia/ or *hemorrhagic shock/ or *traumatic shock/ or exp *bleeding/ or *exsanguination/ |
| 3. | (h?emorrhag* or hypovol?em* or shock or exsanguin* or olig?em* or hypoperfus*).ti,ab. |
| 4. | (bleed* or bloodloss*).ti,ab. |
| 5. | (blood* adj3 loss*).ti,ab. |
| 6. | (coagulopath* or (abnormal* adj2 coagulation) or hyperfibrinolysis).ti,ab. |
| 7. | or/2-6 |
| 8. | exp *blood transfusion/ |
| 9. | ((red blood cell* or rbc or prbc or red cell* or blood or packed cell* or erythrocyte* or fluid* or volum*) adj3 (therap* or transfus* replac* or resuscita* or substitut* or restor* or deficien* or replenish*)).ti,ab. |
| 10. | exp *plasma/ |
| 11. | exp *blood component therapy/ |
| 12. | exp *erythrocyte transfusion/ |
| 13. | (ffp or ((frozen or fresh or thawed or tp) adj3 plasma)).ti,ab. |
| 14. | ((lyophilised or freeze-dried or liquid or "not frozen" or "never frozen") adj 2 plasma).ti,ab. |
| 15. | (fdsp or fdp or lqp or lhp).ti,ab. |
| 16. | exp *freeze drying/ and plasma.ti,ab,sh. |
| 17. | exp *sodium chloride/ |
| 18. | exp *fluid therapy/ |
| 19. | exp *rehydration solutions/ |
| 20. | exp *plasma substitutes/ |
| 21. | exp *isotonic solutions/ |
| 22. | (sodium or salin* or hartman* or ringer* or lactate* or acetate* or plasmalyte* or plasmalyte*).ti,ab. |
| 23. | (crystalloid* or isotonic).ti,ab. |
| 24. | exp *crystalloid/ |
| 25. | ((balanced or physiologic*) adj2 (fluid* or solution*)).ti,ab. |
| 26. | or/8-25 |
| 27. | 1 and 7 and 26 |

**Cochrane search terms**

| #1. | *standard trauma population (see F.2.1)* |
| --- | --- |
| #2. | MeSH descriptor: [hemorrhage] this term only |
| #3. | MeSH descriptor: [exsanguination] this term only |
| #4. | MeSH descriptor: [shock] this term only |
| #5. | MeSH descriptor: [shock, traumatic] this term only |
| #6. | MeSH descriptor: [shock, hemorrhagic] this term only |
| #7. | MeSH descriptor: [hypovolemia] this term only |
| #8. | (haemorrhag* or hemorrhag* or hypovolem* or hypovolaem* or shock or exsanguin* or oligem* or oligaem* or hypoperfus*):ti,ab |
| #9. | (coagulopath* or (abnormal* near/2 coagulation) or hyperfibrinolysis):ti,ab |
| #10. | (bleed* or bloodloss*):ti,ab |
| #11. | blood* near/3 loss*:ti,ab |
| #12. | {or #2-#11} |
| #13. | ((red blood cell* or rbc or prbc or red cell* or blood or packed cell* or erythrocyte*or fluid* or volum* or plasma*) near/5 (therap* or transfus* or replac* or resuscita* or substitut* or restor* or deficien* or replenish*)):ti,ab |
| #14. | MeSH descriptor: [plasma] explode all trees |
| #15. | ((ffp or frozen or thawed or tp or fresh) near/3 plasma):ti,ab |
| #16. | (albumin or zenalb or octaplas*) .ti,ab. |
| #17. | ((lyophili?ed or freeze-dried or liquid or "not frozen" or "never frozen") near/3 plasma):ti,ab |
| #18. | (fdsp or fdp or lqp or lhp):ti,ab |
| #19. | ((balanced or physiologic*) near/2 (fluid* or solution*)):ti,ab |
| #20. | (crystalloid* or isotonic):ti,ab |
| #21. | (sodium or salin* or hartman* or ringer* or lactate* or acetate* or plasmalyte* or plasmalyte*):ti,ab |
| #22. | MeSH descriptor: [freeze drying] explode all trees |
| #23. | MeSH descriptor: [sodium chloride] explode all trees |
| #24. | MeSH descriptor: [fluid therapy] explode all trees |
| #25. | MeSH descriptor: [rehydration solutions] explode all trees |
| #26. | MeSH descriptor: [plasma substitutes] explode all trees |
| #27. | MeSH descriptor: [isotonic solutions] explode all trees |
| #28. | {or #13-#27} |
| #29. | #1 and #12 and #28 |
| #30. | MeSH descriptor: [blood transfusion] this term only |
| #31. | MeSH descriptor: [blood component transfusion] explode all trees |
| #32. | MeSH descriptor: [exchange transfusion, whole blood] this term only |
| #33. | MeSH descriptor: [plasma exchange] this term only |
| #34. | {or #30-#33} |
| #35. | #29 or #34 |

# Table S1: Detailed general characteristics of included systematic reviews

| **Authors, years** | **Research questions/ hypothesis** | **Databases searched** | **Study design,**  **Years**  **searched** | **N° of included studies/ N° of participants** | **Quality assessment** | **Setting** | **Interventions/**  **comparator** | **Outcomes** | **Main findings (narrative)** |
| --- | --- | --- | --- | --- | --- | --- | --- | --- | --- |
| **Comparison 1:** Hypertonic Crystalloids vs isotonic crystalloids | | | | | | | | | |
| Safiejko 2020 | To assess the effect of hypertonic  saline/dextran or hypertonic saline for fluid resuscitation on patient outcomes restricted to  adults with hypovolemic shock | PubMed, Embase, Web of  Science, and the Cochrane library | RCT  Search up to August 20th 2020 | Fifteen studies including 3264 patients | Criteria of the Cochrane risk-of-bias tool for trials | Prehospital / ED | Hypertonic saline/dextran or hypertonic saline versus isotonic fluid | -Short‐ term survival (hospital discharge or 28 to 30 days).  - long‐ term mortality (≥ 3 months), 24-hour mortality, overall, mortality,  -adverse outcome,  -length of stay in an intensive care unit and hospital,  -laboratory parameters at patient admission,  -the Glasgow Outcome Scale Extended score | This systematic review and meta-analysis did not  result in superior 28- to 30-day survival as well as in survival to hospital discharge. Patients with hypotension who received resuscitation with hypertonic saline/dextran had less  overall mortality as patients who received conventional fluid |
| **Comparison 2a:** Use of blood components | | | | | | | | | |
| Coccolini 2019 | To evaluate the effects of plasma in the management of patients suffering  from haemorrhagic shock | MEDLINE,  Embase, PubMed, Cochrane Library and CINAHL | RCT  Search up to August 2018 | Two studies including 626 patients | Criteria of the Cochrane risk-of-bias tool for trials | Prehospital | pre-hospital plasma versus usual care | - 24-hours mortality  - Late mortality (within 30 days)  - morbidity (acute lung injury and multi-organ failure) | Pre-hospital infusions of plasma can reduce 24 hours mortality in haemorrhagic shock patients. It does not  seem to influence 1-month mortality, acute lung injury and multi-organ failure rates. |
| Rijnhout 2019 | To systematically review the evidence for effectiveness and safety of PHBT to haemorrhagic  trauma patients | CINAHL, Cochrane, Embase, and PubMed | RCT and retrospective cohort studies,  Search up to August 1st 2018 | Nine studies including 3212 patients | Criteria of the Cochrane risk-of-bias tool for trials and Risk Of Bias In Non-  randomised Studies of Interventions | Prehospital | Prehospital blood-component transfusion (PHBT) versus standard care | - 24-hours mortality  - Late mortality (within 30 days)  - adverse events | pRBCs and plasma used simultaneously with PHBT resulted in a significant reduction in  the probability for long-term mortality. The authors could not affirm any conclusion about a possible survival benefit for haemorrhagic trauma patients receiving PHBT due to poor quality of included studies. |
| **Comparison 2b:** Blood components ratio | | | | | | | | | |
| Kleinveld 2021 | High platelet:RBC ratios are associated with lower early mortality  when compared with lower platelet:RBC ratios, but have  similar effects on the occurrence of organ failure and  thrombosis. | Medline, PubMed, and Embase | RCT, from 1946 until October 2020 | Five studies including 1757 patients | Criteria of the Cochrane risk-of-bias tool for trials | Prehospital | High platelet:RBC ratios (highest comparator arm) versus lower platelet:RBC ratios (lowest comparator arm) | - 24-hours mortality  - Late mortality (within 30 days)  - thromboembolic events  - organ failure  - correction of coagulopathy (a normalization of coagulation test results within 24 h after transfusion) | High platelet:  RBC compared with a low platelet:RBC ratio significantly improved 24-hour mortality and 30-day mortality. There was no difference between platelet:RBC ratio groups in thromboembolic events, organ failure and correction of coagulopathy. |
| Luz 2019 | To compare the effects of high transfusion ratio of FFP and/or  PLTs to RBCs to low ratios on in-hospital mortality, exposure to allogeneic blood products, and coagulopathy | Medline, Embase, Cochrane Controlled Trials Register, ClinicalTrials.gov, and Google Scholar | RCT and observational studies up to July 31, 2018 | Fifty-five studies including 27133 patients | Criteria of the Cochrane risk-of-bias tool for trials and the Newcastle–Ottawa Scale | Not reported | High fixed transfusion ratio of FFP and PLTs to RBCs or FFP or PLTs to RBCs versus a low fixed transfusion ratio of FFP and PLTs to RBCs or FFP or RBCs to RBCs | - 24-hours hospital mortality  - Late mortality (within 30 days)  - cumulative number of allogeneic RBCs, FFP, and PLT units transfused in 24 hours postadmission  - effect on the ATC, fibrinogen, and TEG or ROTEM variables | Meta-analyses in observational  studies suggest survival benefit and no difference in  exposure to ABPs. No survival benefit in RCTs was  identified. |
| McQuilten 2018 | To assess the effect of dose,  timing and ratio of blood component therapy (FFP, platelets,  cryoprecipitate or fibrinogen concentrate) to RBCs on morbidity, mortality  and transfusion in critically bleeding patients | MEDLINE, Embase, CINAHL, the Cochrane library and the Transfusion Evidence Library | RCT; search up to 21st February 2017 | Six studies, including 987 patients | Criteria of the Cochrane risk-of-bias tool for trials | Not reported | High FFP: RBC ratio versus low FFP: RBC ratio | - 24-hours mortality  - Late mortality (within 28 days)  - morbidity  - length of stay (hospital and ICU)  - transfusion-related adverse events  - correction of coagulopathy  - hospital readmissions  - quality of life  - costs | Higher transfusion ratios were associated with transfusion of more FFP and platelets without  evidence of significant difference with respect to mortality or morbidity. |
| Rahouma 2018 | To compare the effect of balanced, massive transfusion on mortality  and secondary outcomes including ARDS and ALI in a large, cohort of  surgical patients, including both trauma and non-trauma sub  specialties | PubMed, MEDLINE, EMBASE, Web of  Science, Science Direct, and Google scholar | RCT and observational studies up to January 10th, 2016 | Thirty-six studies including 17187 patients | Newcastle-  Ottawa Scale | Twenty-eight studies were from civilian hospitals, 4 from military settings, and 4 from combined civilian/military settings | High FFP: RBC ratio versus low FFP: RBC ratio | - 24-hours mortality  - Late mortality (within 30 days)  - mortality differences in trauma vs. non-trauma patients  - mortality differences  around the different cut-off ratios (1:1, 1:1.5, or 1:2)  - ALI and ARDS  differences between high vs. low ratios | High FFP:RBC ratio confers survival benefits in trauma and non-trauma settings, with the  highest survival benefit at 1:1.5. |
| Ritchie 2020 | What is the most effective empirical transfusion strategy to manage exsanguinating adult trauma patients? | EMBASE, MEDLINE, web of science and  CINAHL | RCT; search up to 10th May 2019 | Seven studies, including 1106 patients | Scottish Intercollegiate Guidelines Network  checklist for RCTs and Criteria of the Cochrane risk-of-bias tool for trials | Emergency department | 2:1 ratio of packed red cell to fresh frozen plasma, standard protocol with fibrinogen or whole blood transfusion versus placebo or standard therapy | - Mortality  - hospital length of stay  - complications  - total transfusion requirements | Early administration of cryoprecipitate is associated  with a lower risk of mortality. There are no differences regarding morbidity |
| Rijnhout 2021 | A PLT/RBC ratio, which approximates the content of (fresh) whole blood, will result  in a survival benefit, as compared with a lower PLT/RBC ratio | PubMed, CINAHL, Embase, and Cochrane | RCT and observational studies up to January 21,  2021 | Twelve studies including 5118 patients | Criteria of the Cochrane risk-of-bias tool for trials and Risk Of Bias In Nonrandomized Studies-of Interventions tool | Pre-hospital | High PLT/RBC ratio versus low PLT/RBC ratio | - Early Mortality (1 Hour to  6 Hours and 24 Hours)  - 28-Day to 30-Day Mortality  - total amount of RBC  Transfusion  - ICU and Hospital length of stay and Ventilator (Free) Days | Higher PLT/RBC ratios are associated with significantly lower 1-hour to 6-hour, 24-hour, 28-day to 30-day mortalities as compared  with lower PLT/RBC ratios. The optimal PLT/RBC ratio for massive transfusion in trauma patients is approximately 1:1. |
| Wirtz 2020 | To analyse the incidence and risk for developing thromboembolic  events associated with current transfusion and  resuscitation strategies in trauma patients | MEDLINE, PubMed,  and Embase | RCT and observational studies. Years searched not reported | Forty studies including 11074 patients | Criteria of the Cochrane risk-of-bias tool for trials and the Newcastle–Ottawa Scale | Not reported | High plasma-to-RBC ratio versus low plasma-to-RBC ratio | - Development of  thromboembolic events in trauma patients | The authors identified an  incidence of thromboembolic events of 10% in severely  injured bleeding trauma patients. The use of tranexamic acid and fibrinogen concentrate was associated with the  development of thromboembolic complications. |
| **Comparison 3:** Whole blood vs component therapy | | | | | | | | | |
| Avery 2020 | To establish if there is an improvement in  survival at 30 days with the use of WB transfusion  compared with blood component therapy in adult trauma patients with acute major haemorrhage. | PubMed, Web of Science, Cochrane, OVID,  Embase and the Transfusion Evidence Library | RCT and observational studies up to 15th December 2019 | Six studies including 3255 patients | Cochrane risk of bias tool | Pre-hospital | Use of WB transfusion versus blood component therapy | - Late mortality (within 30 days  - 24-hours mortality  - total volume of transfusion  - morbidity (acute respiratory distress syndrome and acute kidney injury, | No evidence provided supported or rejected use of WB transfusion  compared with component therapy for adult trauma  patients with acute major haemorrhage, due to poor-quality studies included. |
| **Crowe 2020** | To determine the association of whole blood with mortality  after traumatic haemorrhagic shock. | MEDLINE, Embase, and the Cochrane Library | RCT and observational studies up to June 2019 | Twelve studies including 8431 patients | Newcastle-Ottawa Scale | Pre-hospital | Whole blood transfusion compared with component therapy | - 24-hours mortality  - Late mortality (within 30 days)  - in-hospital mortality | Whole blood was not associated with 24-hour or in-hospital  mortality. |
| Cruciani 2020 | To assess the efficacy and safety of WB  in trauma-associated massive bleeding | PubMed, Embase, Scopus, Ovid and  Cochrane Library | RCT and observational studies up to March 6, 2020 | Eight studies including 3642 patients | Criteria of the Cochrane risk-of-bias tool for trials and Risk Of Bias In Nonrandomized Studies-of Interventions tool | Pre-hospital/ Emergency Department | Whole blood in massive trauma  Bleeding versus transfusion of blood component | - 24-hours mortality  - Late mortality (within 30 days)  - adverse events/ transfusion reactions | 30-day/in-hospital and 24-h  mortality did not differ significantly between groups. The available evidence does not allow to draw definite conclusions on the short-term and long-term efficacy and safety of WB transfusion compared to blood components transfusion |
| Malkin 2020 | To evaluate the efficacy and safety of resuscitation with WB, compared to resuscitation with blood components, in haemorrhaging trauma patients. | MEDLINE, Embase and ISI Web of Science | RCT and observational studies up to 2018 | Five studies including 1292 patients | Criteria of the Cochrane risk-of-bias tool for trials and Risk Of Bias In Nonrandomized Studies-of Interventions tool | Pre-hospital | WB resuscitation compared to resuscitation with blood components in balanced ratios | - 24-hours mortality  - Late mortality (within 30 days)  - blood product utilization  - adverse events | WB was not associated with a significant survival benefit or reduced blood product utilization. The overall quality of studies was poor |
| McQuilten 2018 | To assess the effect of dose,  timing and ratio of blood component therapy (FFP, platelets,  cryoprecipitate or fibrinogen concentrate) to RBCs on morbidity, mortality  and transfusion in critically bleeding patients | MEDLINE, Embase, CINAHL, the Cochrane library and the Transfusion Evidence Library | RCT; search up to 21st February 2017 | Six studies. Number including 987 patients | Criteria of the Cochrane risk-of-bias tool for trials | Not reported | WB versus component therapy | - 24-hours mortality  - Late mortality (within 28 days)  - morbidity  - length of stay (hospital and ICU)  - transfusion-related adverse events  - correction of coagulopathy  - hospital readmissions  - quality of life  - costs | Higher transfusion ratios were associated with transfusion of more FFP and platelets without  evidence of significant difference with respect to mortality or morbidity. |
| **Ritchie 2020** | What is the most effective empirical transfusion strategy to manage  exsanguinating adult trauma patients? | EMBASE, MEDLINE, web of science and  CINAHL | RCT; search up to 10th May 2019 | Seven studies, including 1106 patients | Scottish Intercollegiate Guidelines Network  checklist for RCTs and Criteria of the Cochrane risk-of-bias tool for trials | Emergency department | 2:1 ratio of packed red cell to fresh frozen plasma, standard protocol with fibrinogen or whole blood transfusion versus placebo or standard therapy | - Mortality  - hospital length of stay  - complications  - total transfusion requirements | Early administration of cryoprecipitate is associated  with a lower risk of mortality. There are no differences regarding morbidity |
| **Notes**. ABPs: allogeneic blood products; ALI: acute lung injury; ARDS: acute respiratory distress syndrome; ATC: acute traumacoagulopathy FFP= fresh frozen plasma; ICU: intensive care unit PHBT: prehospital blood-component transfusion PLT: platelets; pRBCs: packed red blood cells RBC: red blood cells; ROTEM rotational thrombelastometry TEG: thromboelastography WB: whole blood; | | | | | | | | | |

# Table S2: Map of primary studies contained within each included systematic review per different comparison

| **Comparison 1. Hypertonic crystalloids vs isotonic crystalloids** | *Systematic review* | |
| --- | --- | --- |
| *Primary studies* | | Safiejko 2020 |
|  | |  |
| Bulger 2011 | | x |
| Morrison 2011 | | x |
| Rizoli 2006 | | x |
| Vassar 1991 | | x |
| Vassar 1993 (1) | | x |
| Vassar 1993 (2) | | x |
| Wade 2003 | | x |
| Younes 1992 | | x |
| Cooper 2004 | | x |
| Holcroft 1987 | | x |
| Bulger 2008 | | x |
| Holcroft 1989 | | x |
| Younes 2002 | | x |
| Alpar 2004 | | x |
| Mattox 1991 | | x |

| Comparison 2a: Use of blood components | *Systematic reviews* | |
| --- | --- | --- |
| *Primary studies* | Rijnhout 2019 | Coccolini 2019 |
|  |  |  |
| Miller 2016 | x |  |
| Peters 2017 | x |  |
| Rehn 2018 | x |  |
| Brown 2015 | x |  |
| Shackelford 2017 | x |  |
| Holcomb 2017 | x |  |
| O'reilly 2014 | x |  |
| Sperry 2018 | x | x |
| Moore 2018 | x | x |

| **Comparison 2b: Blood component ratios** | | | | | | | | |
| --- | --- | --- | --- | --- | --- | --- | --- | --- |
|  | | | | | *Systematic reviews* | | | |
|  | MC Quinten 2020 | Luz 2019 | Ritchie 2020 | Rahouma 2016 | | Wirtz 2019 | Rijnhout 2021 | Kleinveld 2020 |
| PLT: PRBC ratio | | | | | | | | |
| *Primary studies* |  |  |  |  | |  |  |  |
|  |  |  |  |  | |  |  |  |
| Baksaas -Aasen 2020 |  |  |  |  | |  |  | x |
| Gonzales 2016 |  |  |  |  | |  | x | x |
| Lusterberger 2011 |  |  |  |  | |  | x |  |
| Shaz 2010 |  | x |  |  | |  | x |  |
| Rowel 2012 |  |  |  |  | |  | x |  |
| Inaba 2010 |  |  |  |  | |  | x |  |
| Cap 2012 |  |  |  |  | | x | x |  |
| Balvers 2017 |  | x |  |  | |  | x |  |
| Brown 2012 |  | x |  |  | |  | x |  |
| Holcomb 2011 |  | x |  |  | |  | x |  |
| Perkins 2012 |  |  |  |  | |  | x |  |
| Nascimento 2013 |  |  |  |  | |  | x | x |
| Holcomb 2015 |  |  |  |  | |  | x | x |
| Sperry 2018 |  |  |  |  | |  |  | x |
| FFP: RBC ratio | | | | | | | | |
| Van 2010 |  | x |  | x | |  |  |  |
| Snyder 2009 |  | x |  | x | |  |  |  |
| Teixeira 2009 |  | x |  | x | |  |  |  |
| Dente 2009 |  | x |  | x | |  |  |  |
| Sharpe 2012 |  | x |  | x | |  |  |  |
| Spinella 2011 |  | x |  | x | |  |  |  |
| Peiniger 2011 |  | x |  | x | |  |  |  |
| Magnotti 2011 |  | x |  | x | |  |  |  |
| Stanworth 2016 |  | x |  | x | |  |  |  |
| Borgman 2011 |  | x |  | x | |  |  |  |
| Brown 2011 |  | x |  | x | |  |  |  |
| Shaz 2010 |  | x |  | x | |  |  |  |
| Rowel 2011 |  | x |  | x | |  |  |  |
| Kashuk 2008 |  | x |  | x | |  |  |  |
| Nardi 2015 |  | x |  |  | |  |  |  |
| Mazzeffi 2016 |  |  |  | x | |  |  |  |
| Mell 2010 |  |  |  | x | |  |  |  |
| Kim 2014 |  | x |  | x | |  |  |  |
| Holcomb 2008 |  | x |  | x | |  |  |  |
| Brown 2012 |  | x |  |  | |  |  |  |
| Mitra 2010 |  |  |  | x | |  |  |  |
| Sperry 2008 |  | x |  | x | |  |  |  |
| Lustenberger 2011 |  | x |  | x | |  |  |  |
| Handin 2014 |  | x |  | x | |  |  |  |
| Borgaman 2007 |  | x |  | x | |  |  |  |
| Kudo 2013 |  | x |  |  | |  |  |  |
| Bui 2016 |  | x |  |  | |  |  |  |
| Spoerke 2011 |  |  |  | x | |  |  |  |
| Balvers 2017 |  | x |  |  | |  |  |  |
| Maegele 2008 |  | x |  | x | |  |  |  |
| Zink 2009 |  | x |  | x | |  |  |  |
| Vulliamy 2017 |  | x |  |  | |  |  |  |
| Wafaisade 2011 |  | x |  | x | |  |  |  |
| Sambasivan 2011 |  | x |  |  | |  |  |  |
| Perkins 2009 |  | x |  |  | |  |  |  |
| Holcom 2011 |  | x |  |  | |  |  |  |
| Haltmeier 2017 |  | x |  |  | |  |  |  |
| Sharpe 2012 |  |  |  |  | |  |  |  |
| Duchesne 2009 |  | x |  | x | |  |  |  |
| Undurraga 2015 |  |  |  | x | |  |  |  |
| Duchesne 2008 |  | x |  |  | |  |  |  |
| FFP: PLT: PRBC ratio | | | | | | | | |
| Nascimento 2013 | x | x | x |  | |  |  |  |
| Holcomb 2015 | x | x | x |  | |  |  |  |

| **Comparison 3: Whole Blood component** **vs component therapy** | | | | | | | |
| --- | --- | --- | --- | --- | --- | --- | --- |
|  | | | | *Systematic reviews* | | | |
|  | MC Quinten 2020 | Crowe 2020 | Avery 2020 | | Cruciani 2020 | Malkin 2020 | Ritchie 2020 |
| *Primary studies* |  |  |  | |  |  |  |
|  |  |  |  | |  |  |  |
| Cotton, 2013 | x | x | x | | x | x | x |
| Williams 2019 |  | x |  | | x |  |  |
| Zhu 2019 |  | x |  | |  |  |  |
| Seheult 2018 |  | x |  | |  | x |  |
| Yazer 2016 |  | x |  | |  |  |  |
| Auten 2015 |  | x |  | |  |  |  |
| Keneally 2015 |  | x |  | |  |  |  |
| Jones 2014 |  | x |  | | x |  |  |
| Nessen 2013 |  | x |  | | x |  |  |
| Ho 2011 |  | x |  | | x |  |  |
| Perkins 2011 |  | x | x | | x | x |  |
| Spinella 2009 |  | x | x | | x | x |  |
| Rahbar 2015 |  |  |  | |  |  | x |
| Holcomb 2015 |  |  |  | |  |  | x |

# Table S3. Mortality/survival, comparison hypertonic crystalloids vs isotonic crystalloids

| **Author – year** | **Study design** | **Mortality at 24 hours, 30 days/1month and 12 months** |
| --- | --- | --- |
| ***Hypertonic saline/dextran or hypertonic saline versus isotonic fluid*** | | |
| **Safiejko 2020** | SR (15 RCTs) | **24 h survival** (Holcroft 1987, Morrison 2011, Rizoli 2006, Vassar 1991, Vassar 1993 (1), Vassar 1993 (2), Wade 2003, Younes 1992, Cooper 2004)  Survival to hospital discharge rate 88.6% in hypertonic saline/dextran (HSD) group vs. 72.3% for isotonic fluid (NS) solutions (OR = 2.99; 95% CI 2.04–4.39; I2 = 0%; p = 0.001).  **28- to 30-days survival rate** (Bulger 2011, Bulger 2008, Holcroft 1989, Morrison 2011, Younes, 2002)  Pooled analysis showed that the use of hypertonic fluid solutions was 72.8% survivable, while in the case of isotonic fluid (NS) (OR = 1.13; 95% CI 0.75–1.70; I2 = 54%; p = 0.56).  Subgroup:   - *hypertonic saline/dextran (HSD) vs isotonic fluid (NS) solutions*: OR 1.06 (95% CI 0.64 – 1.77, I2=56%, p=0.08; 4 trials); - *hypertonic saline vs isotonic fluid (NS) solutions:* OR 1.14 (95% CI 0.71 – 1.83, I2=49%, p=0.16; 2 trials).   **Overall mortality** (Alpar 2004, Bulger 2011, Mattox 1991, Rizoli 2006, Wade 2003, Younes 1992, Younes 2002)  Overall, OR 0.76 (95% IC 0.61 – 0.94) I2=33%; p=0.01.  Subgroup:   - *hypertonic saline/dextran (HSD) vs isotonic fluid (NS) solutions*: OR 0.72 (95% CI 0.55 – 0.94, I2=20%, p=0.28; 6 trials); - *hypertonic saline vs isotonic fluid (NS) solutions:* OR 0.90 (95% CI 0.66 – 1.23, I2=10%, p=0.33; 3 trials) |

| **Author - year** | **Study design** | **Mortality at 24 hours, 30 days/1month and 12 months** | |
| --- | --- | --- | --- |
| ***PRBCs versus standard care*** | | |  |
| **Rijnhout 2019** | SR (7 observationals) | **Mortality 24 h** (Miller 2016, Peters 2017, Rehen 2018)  OR = 0.92; 95% CI, 0.46–1.85; P = 0.82, I2=80%  **Mortality 30 days** (Brown 2015, Miller 2016, Peters 2017, Rehen 2018)  OR = 1.18; 95% CI, 0.93–1.49; P = 0.17, I2=18% | |
| ***PRBCs + plasma versus standard care*** | | |  |
| **Rijnhout 2019** | SR (1 RCT, 3 observationals) | **Mortality 24 h** (Shackelford 2017, Holcomb 2017)  OR = 0.47; 95% CI, 0.17–1.34; I2=48% P = 0.16  **Mortality 30 days** (Sperry 2018, O’reilly 2014, Shackelford 2017, Holcomb 2017)  OR = 0.51; 95% CI, 0.33–0.81; P < 0.0001 (1 RCT)  OR = 0.49; 95% CI, 0.29–0.83 P = 0.008; i2=0% (3 observationals) | |
| ***plasma versus standard care*** | | |  |
| **Rijnhout 2019** | SR (1 RCT) | **Mortality 24 h** **– 1 month** (Moore 2018)  More patients in the plasma group died than in the control group, but not significantly 24 h (p=0.68) and 1 month (p=0.37) | |
| **Coccolini 2019** | SR (2 RCTs) | **Mortality 24 h** (Moore 2018, Sperry 2018)  RR = 0.69; 95% CI = 0.48–0.99; i2=34%  **Mortality 1 month** (Moore 2018, Sperry 2018)  RR = 0.86; 95% CI = 0.68–1.11; i2=38% | |

# Table S4. Mortality, use of blood components.

# Table S5. Mortality, blood components ratios.

| Author - year | Study design | Mortality at 24 hours, 30 days/1month and 12 months | |
| --- | --- | --- | --- |
| 1. FFP: PLT: PRBC ratio | | | |
| McQuilten 2018 | SR (2 RCTs) | **Mortality 24 h** (Holcomb 2015)  RR 0.75(0.52-1.08)  Anticipated absolute effects control 170 per 1000, intervention 42 fewer per 1000 (81 fewer to 14 more).  **Mortality 28 days** (Nascimento 2013, Holcomb 2015)  The fixed effects pooled RR 1.26 (0.49-3.22), for 28-days mortality, there was a moderate/high level of heterogeneity (I2 = 75%, P = 0.64). | |
| Luz 2019 | SR (2 RCTs) | **Mortality 24 h** (Holcomb 2015)  Quantitative sysntesis: There was a lower rate of death from exsanguination at 24 hours (9.2% in 1:1:1 vs. 14.6% in 1:1:2 group; difference: -5.4% (95% CI -10.4% to -0.5%]; p = 0.03)  **Mortality 30 days** (Nascimento 2013, Holcomb 2015)  The fixed effects pooled OR 1.35 (0.40-4.59), for 30-days mortality, there was a moderate/high level of heterogeneity (I2 = 76%, P = 0.63) (Figure 2). | |
| Ritchie 2020 | SR (2 RCTs) | **Mortality 24 h** (Holcomb et al. 2015)  RR 1.33 (0.93 – 1.92)  **Mortality 1 month** (Holcomb et al. 2015)  RR 1.17 (0.90 – 1.53) | |
| 1. FFP: RBC ratios | | | |
| Luz 2019*  *some studies of the meta-analysis with wrong intervention and/or comparator | SR | FFP:RBC 1:1 vs <1:1  5 observational studies  10 observational studies  FFP:RBC 1:1.5 vs <1:1.5  2 observational studies  1 RCT, 4 observational studies  FFP:RBC 1:2 vs <1:2  6 observational studies  10 observational studies | **Mortality 24 h** (Balvers 2017, Perkins 2009, Maegele 2008, Vulliamy 2017, Wafaisade 2011) 2414 participants  The random effects pooled OR 0.34 (95% confidence [CI] = 0.14–0.82). For 24-hour mortality, there was a high level of heterogeneity (I2 = 88%, P < 0.01).  **Mortality 30 days** (Duchesne 2009, Duchesne 2008, Holcom 2011, Haltmeier 2017, Perkins 2009, Maegele 2008, Zink 2009, Vulliamy 2017, Wafaisade 2011, Sambasivan 2011) 4203 participants  The random effects pooled OR 0.38 (95% confidence [CI] = 0.22–0.68). For 30 days mortality, there was a high level of heterogeneity (I2 = 91%, P <0.01).  **Mortality 24 h** (Kudo 2013, Bui 2016)118 participants  The random effects pooled OR 0.43 (95% confidence [CI] = 0.18–1.06). For 24-hour mortality, there was an absent level of heterogeneity (I2 = 0%, P =0.41).  **Mortality 30 days** (Lustenberger 2011, Handin 2014, Borgaman 2007, Kudo 2013, Sperry 2008) 1369 participants  The random effects pooled OR 0.42 (95% confidence [CI] = 0.22–0.81). For 30 days mortality, there was a moderate/high level of heterogeneity (I2 = 73%, P =0.005).  **Mortality 24 h** (Kim 2014, Nardi 2015, Rowel 2011, Snyder 2009, Stanworth 2016) 1388 participants  The random effects pooled OR 0.59 (95% confidence [CI] = 0.43–0.81). For 24-hour mortality, there was a low level of heterogeneity (I2 = 22%, P =0.27).  **Mortality 30 days** (Borgman 2011, Holcomb 2008, Magnotti 2011, Nardi 2015, Kim 2014, Peiniger 2011, Sharpe 2012, Teixeira 2009, Rowel 2011, Van 2010) 2849 participants  The random effects pooled OR 0.47 (95% confidence [CI] = 0.31–0.71). For 30 days mortality, there was a high level of heterogeneity (I2 = 81%, P <0.01). |
| Rahouma 2016*  *some studies of the meta-analysis with wrong intervention and/or comparator | SR | FFP:RBC <1:1 vs ≥1:1  6 observational studies and 1 RCT  5 observational studies and 2 RCTs  FFP:RBC <1:1.5 vs ≥1:1.5  1 RCT, 3 observational studies  1 RCT, 4 observational studies  FFP:RBC <1:2 vs ≥1:2  9 observational studies  14 observational studies | **Mortality 24 h** (Holcomb 2015, Duchesene 2009, Maegele 2008, Spoerke 2011, Sharpe 2012, Undurraga 2015, Wafaisade 2011) 5265 participants  The random effects pooled OR 2.05 (95% confidence [CI] = 1.55–2.71). For 24-hour mortality, there was a moderate level of heterogeneity (I2 = 57%, P = 0.03).  **Mortality 30 days** (Holcomb 2015, Maegele 2008, Nascimento 2013, Spoerke 2011, Undurraga 2015, Zink 2009, Wafaisade 2011) 5266 participants  The random effects pooled OR 1.36 (95% confidence [CI] = 1.09–1.69). For 30 days mortality, there was a low/moderate level of heterogeneity (I2 = 45%, P =0.09).  **Mortality 24 h** (Sperry 2008, Lustenberger 2011, Handin 2014, Mitra 2010) 1877 participants  The random effects pooled OR 3.97 (95% confidence [CI] = 1.37–11.49). For 24-hour mortality, there was a high level of heterogeneity (I2 = 88%, P <0.01).  **Mortality 30 days** (Borgaman 2007, Brown 2012, Lustenberger 2011, Mitra 2010, Sperry 2008**)** 1813 participants  The random effects pooled OR 2.45 (95% confidence [CI] = 1.14–5.25). For 30 days mortality, there was a high level of heterogeneity (I2 = 87%, P <0.01).  **Mortality 24 h** (Borgman 2011, Dente 2009, Kashuk 2008, Kim 2014, Peiniger 2011, Magnotti 2011, Shaz 2010, Rowel 2011, Stanworth 2014) 3540 participants  The random effects pooled OR 2.85 (95% confidence [CI] = 2.14–3.81). For 24-hour mortality, there was a moderate level of heterogeneity (I2 = 59%, P =0.01).  **Mortality 30 days** (Borgman 2011, Brown 2011, Holcomb 2008, Duchesene 2009, Mazzetti 2016, Mell 2010, Kim 2014, Rowel 2011, Shaz 2010, Peiniger 2011, Spinella 2011, Van 2010, Snyder 2009, Teixeira 2009) 6193 participants  The random effects pooled OR 1.77 (95% confidence [CI] = 1.50–2.10). For 30 days mortality, there was a low level of heterogeneity (I2 = 37%, P =0.08). |
| C. PLT:PRBC ratio | | | |
| Luz 2019 | SR | HIGH (≥1:1) vs LOW (<1:1)  1 observational  HIGH (≥1:9) vs LOW (<1:9)  1 observational  LOW (> 1:20) vs HIGH (1:1)  1 observational  MEDIUM (1:2) vs HIGH (1:1)  1 observational  HIGH (1:8), MEDIUM (1:16 to 1:8), LOW (< 1:16)  1 observational  HIGH (≥1:2) vs LOW (<1:2)  2 observational | **Survival and free of massive transfusion 24 h (**Balvers 2018)  OR 2.67 (1.24 – 5.77)  **Mortality 24 h (**Brown 2012)  - PLT:PRBC ratio within 6 h: HR 0.34 (0.11 – 0.89)  - PLT:PRBC ratio within 12 h: HR 0.17 (0.04 – 0.54)  - PLT:PRBC ratio within 24 h: HR 0.26 (0.06 – 0.86)  **Mortality 24 h (**Holcomb 2011)  RR 2.81 (1.36 – 5.8)  **Mortality at 30 days (**Holcomb 2011)  RR 1.77 (1.16 – 2.68)  **Mortality 24 h** (Holcomb 2011)  RR 3.13 (1.52 – 6.45)  **Mortality at 30 days (**Holcomb 2011)  RR 1.75 (1.15 – 2.65)  **Mortality 24 h** (Perkins 2009)  OR 0.82 (0.72 – 0.93)  **Mortality 30 days** (Perkins 2009)  OR 0.91 (0.86 – 0.95)  **Survival 30 days** (Shaz 2010)  OR 1.55 (1.09 – 2.18)  **Mortality 30 days** (Spinella 2011)  HR 0.63 (0.39 – 0.86) |
| Wirtz 2019 | SR | HIGH (≥0.1) vs LOW (≤0.1)  1 observational | **Survival 24 h** (Cap 2012)  HR 4.25 (1.25 – 14.48)  **Survival at 30 days** (Cap 2012)  HR 2.32 (1.11 – 4.48) |
| Rijnhout 2021 | SR | HIGH (≥0.3) vs LOW (<0.3)  2 observationals  HIGH (≥0.5) vs LOW (<0.5)  4 observationals  2 observationals  HIGH (≥1) vs LOW (0.5 or <1)  2 RCT, 1 observational  HIGH (≥1) vs LOW (0.6 or <1)  2 RCT, 1 observationals | **Mortality 24 h** (Lustemberger 2011, Shaz 2010) 413 partecipants  OR, 0.12; 95% CI, 0.08–0.21; p < 0.00001  **Mortality 24 h** (Rowel 2012, Inaba 2010, Cap 2012, Perkins 2012) 2143 partecipants  OR, 0.46; 95% CI, 0.28–0.76; p = 0.002; i2=75%  **Mortality 28 days -1 month** (Rowel 2012, Cap 2012) 1117 partecipants  OR, 0.68; 95% CI, 0.50–0.91; p = 0.01, i2=0%  **Mortality 24 h** (Holcomb 2015, Holcomb 2011, Balvers 2018) 1497 partecipants  OR, 0.81; 95% CI, 0.30–2.19; p= 0.68; i2=93%  **Mortality 28 days -1month** (Nascimento 2013, Holcomb 2015, Holcomb 2011) 1181 partecipants  OR, 0.58; 95% CI, 0.35–0.98; p = 0.04; i2=64% |
| Kleinveld 2020 | SR | HIGH ratio vs LOW ratio  5 RCT  HIGH ratio vs LOW ratio  5 RCT | **Mortality 24 h** (Nascimento 2013, Holcomb 2015, Sperry 2018, Gonzales 2016, Baksaas -Aasen 2020) 1757 partecipants  OR 0.69 [95% CI: 0.53–0.89]; i2=41%  **Mortality 30 days** ((Nascimento 2013, Holcomb 2015, Sperry 2008, Gonzales 2016, Baksaas -Aasen 2020) 1757 partecipants  OR 0.78 [95% CI: 0.63–0.98]; i2=47% |

# Table S6. Mortality, whole blood components

| Author - year | Study design | Mortality at 24 hours, 30 days/1month and 12 months |
| --- | --- | --- |
| Whole blood vs component therapy | | |
| McQuilten 2018 | SR (1 RCT) | **Mortality 24 h** (Cotton 2013)  RR 1.13 (0.37-3.49), Anticipated absolute effects control 96 per 1000, intervention 12 more per 1000 (61 fewer to 239 more).  **Mortality 30 days** (Cotton 2013)  RR 1.42 (0.63-3.19), Anticipated absolute effects control 154 per 1000, intervention 65 more per 1000 (57 fewer to 337 more). |
| Crowe 2020 | SR (1 RCT, 11 observational) | **Mortality 24 h** (Cotton, 2013 Williams 2019, Zhu 2019, Seheult 2018, Yazer 2016, Auten 2015, Keneally 2015, Jones 2014, Nessen 2013, Ho 2011, Perkins 2011, Spinella 2009)  The fixed effects pooled OR 0.83 (95% confidence [CI] = 0.56–1.24). For 24-hour mortality, there was a small to moderate level of heterogeneity (I2 = 27.2%, P = 0.37).  **Mortality 30 days** (Cotton, 2013 Williams 2019, Zhu 2019, Seheult 2018, Yazer 2016, Auten 2015, Keneally 2015, Jones 2014, Nessen 2013, Ho 2011, Perkins 2011, Spinella 2009)  The DerSimonian and Laird random effects pooled OR for in-hospital/30-day mortality was 0.79 (95% CI = 0.49–1.31). Moderate to high degree of heterogeneity (I^2^ = 87.3%, P = 0.37). |
| Avery 2020 | SR (1 RCT, 2 observational) | **Mortality 24 h** (Cotton 2013, Perkins 2011, Spinella 2009)  Qualitative synthesis: Two studies found no statistically significant difference in 24 hours mortality between WB transfusion strategy and blood component therapy (Cotton 2013, Perkins 2011). A third, retrospective study (Spinella 2009) found a statistically significant difference in 24 hours mortality between WB transfusion and blood component therapy (4% vs 12% respectively, p=0.018).30 |
| Cruciani 2020 | SR (1 RCT, 6 observational) | **Mortality 24 h (**Cotton 2013, Perkins 2011, Spinella 2009)  Twenty-four-hour mortality did not differ significantly between ‘whole blood’ and ‘blood component’ recipients (OR 0.80; 95% CIs 0.40/1.59; p = 0.53; I2 = 31%). The results were much the same when the analyses were limited to observational studies alone for 24-h mortality, OR 0.84; 95% CIs 0.33/2.15.  **Mortality 30 days** (Cotton 2013, Jones 2014, Nessen 2013, Ho 2011, Perkins 2011, Spinella 2009, Williams 2019)  The effect size did not differ between ‘whole blood’ and ‘blood component’ recipients (OR 0.90: 95 CIs 0.62/1.30; p = 0.56: I2 = 56%).   - The results were much the same when the analyses were limited to observational studies alone (for 30-day/in-hospital mortality, OR 0.82; 95% CIs 0.56/1.20). In the RCT the OR for 30-days mortality was 1.81 (95% CIs 0.80/4.09; p = 0.15). - After adjustment for baseline covariates, the OR for mortality was significantly lower in ‘whole blood’ recipients compared to ‘blood component’ (OR 0.22; 95% CIs 0.10/0.45); p < 0.001; I2 = 12). - 30-day/in-hospital mortality adjusted HR was not statistically significant between groups (HR, 0.72; 95% CIs 0.45/1.16; p = 0.176; I2 = 0). - 30-day crude mortality was higher in civilian than in military setting (27.5% versus 17.4%; p < 0.0001), independently of the type of transfusion (WB or COMP), but probably in relation to the older age and prevalence of chronic conditions in civilian setting |
| Malkin 2020 | SR (1 RCT, 3 observational) | **Mortality 24 h** (Cotton 2013, Perkins 2011, Spinella 2009, Seheult 2018)  **Mortality 30 days** (Cotton 2013, Perkins 2011, Spinella 2009)  Qualitative synthesis: There is an apparent benefit in blood product utilization with the use of WB across most studies. |
| Ritchie 2020 | SR (1 RCT) | **Mortality 24 h** (Cotton 2013, Holcomb 2015, Rahbar 2015)  Qualitative synthesis: The studies looking at 24-hour mortality all showed reduced mortality in the arm receiving standard therapy over the arm receiving the intervention when relative risks were calculated. However, based on the 95% confidence interval, there was no statistically significant difference between the two groups. |

| Supplement 2 - Characteristics of excluded SRs |  |
| --- | --- |
| **Study** | **Reasons** |
| Bentley 2022 - Does fibrinogen concentrate improve outcomes in major traumatic Haemorrhage? A systematic review | Wrong intervention |
| Cardena 2018 - Platelet transfusions improve hemostasis and survival in a substudy of the prospective, randomized PROPPR trial | No outcome of interest- outcomes already included in SR |
| Crescenzo 2017 - Prehospital hypertonic fluid resuscitation for trauma patients: A systematic review and meta-analysis | Not updated reviewed - Complete overlapping with other reviews |
| Doughty 2018 - Massive transfusion: changing practice in a single Norwegian centre 2002–2015 | Wrong intervention |
| Garrigue 2017 - French lyophilized plasma versus fresh frozen plasma for the initial management of trauma-induced coagulopathy: a randomized open-label trial | Wrong intervention |
| Geneen, 2022 - The Difference in Potential Harms between Whole Blood and Component Blood Transfusion in major Bleeding: A Rapid Systematic Review and Meta-Analysis of RCTs | Wrong population |
| Gu 2020 - Restricted fluid resuscitation improves the prognosis of patients with traumatic hemorrhagic shock | Wrong intervention |
| Hanna 2020 - Nationwide analysis of whole blood hemostatic resuscitation in civilian trauma | No outcome of interest- outcomes already included in SR |
| Harada 2017 - 10-Year trend in crystalloid resuscitation: Reduced volume and lower mortality | Wrong intervention |
| Hazelton 2020 - Cold-stored whole blood: A better method of trauma resuscitation? | No outcome of interest- outcomes already included in SR |
| Heuer 2014 - Prehospital fluid management of abdominal organ trauma patients—a matched pair analysis | Wrong intervention |
| Matsuyama, 2018 - Preoperative fluid restriction for trauma patients with hemorrhagic shock decreases ventilator days | Wrong intervention |
| Poole 2016 - Blood Component Therapy and Coagulopathy in Trauma: A Systematic Review of the Literature from the Trauma Update Group | No outcome of interest- outcomes already included in SR |
| Rodríguez 2020 - Mortality in civilian trauma patients and massive blood transfusion treated with high vs low plasma: red blood cell ratio. Systematic review and meta-analysis | No outcome of interest- outcomes already included in SR |
| Rosenfeld 2019 - Defining massive transfusion in civilian pediatric trauma | Wrong intervention |
| Schreiber 2015 - A controlled resuscitation strategy is feasible and safe in hypotensive trauma patients: results of a prospective randomized pilot trial | Wrong intervention |
| Shea 2020 - The use of low-titer group O whole blood is independently associated with improved survival compared to component therapy in adults with severe traumatic hemorrhage | No outcome of interest- outcomes already included in SR |
| Tucker, 2021 - Outcome measures used in clinical research evaluating prehospital blood component transfusion in traumatically injured bleeding patients: A systematic review | No outcome of interest- narrative report of outcome considered in primary studies |
| Zander 2014 - Does resuscitation with plasma increase the risk of venous thromboembolism? | Wrong intervention |
| Zhang 2018 - Clinical effects of two types of fluid infusion in pre-hospital care for traumatic shock | No outcome of interest- outcomes already included in SR |

# Supplement 3 – Summary of findings

[**Criteria for Downgrading – GRADE Approach** 21](#_Toc98756294)

[**2.1 Packed red blood cells (PRBCs) versus standard care** 23](#_Toc98756295)

[**2.2 Packed red blood cells (PRBCs) + plasma versus standard care** 23](#_Toc98756296)

[**2.3 Plasma versus standard care** 24](#_Toc98756297)

[**2.4 FFP:PLT:PRBC ratio** 25](#_Toc98756298)

[**2.5 FFP:PRBC ratio, 1:1 vs <1:1** 25](#_Toc98756299)

[**2.6 FFP:PRBC ratio, 1:1.5 vs <1:1.5** 26](#_Toc98756300)

[**2.7 FFP:PRBC ratio, 1:2 vs <1:2** 26](#_Toc98756301)

[**2.8 PLT:PRBC ratio, 3.3.1 ≥1:1 vs < 1:1** 27](#_Toc98756302)

[**2.9 PLT:PRBC ratio, ≥1:9 vs < 1:9** 27](#_Toc98756303)

[**2.10 PLT:PRBC ratio, >1:20 vs 1:1** 27](#_Toc98756304)

[**2.11 PLT:PRBC ratio, 1:2 vs 1:1** 28](#_Toc98756305)

[**2.12 PLT:PRBC ratio, 1:8 vs 1:16** 28](#_Toc98756306)

[**2.13 PLT:PRBC ratio, ≥ 1:2 vs <1:2** 29](#_Toc98756307)

[**2.14 PLT:PRBC ratio, ≥0.1 vs ≤0.1** 29](#_Toc98756308)

[**2.15 PLT:PRBC ratio, ≥0.3 vs <0.3** 30](#_Toc98756309)

[**2.16 PLT:PRBC ratio, ≥0.5 vs <0.5** 30](#_Toc98756310)

[**2.17 PLT:PRBC ratio, ≥1 vs 0.5 or < 1** 31](#_Toc98756311)

[**2.18 PLT:PRBC ratio, ≥1 vs 0.6 or < 1** 31](#_Toc98756312)

[**2.19 PLT:PRBC ratio, HIGH vs LOW** 31](#_Toc98756313)

[**Comparison 3: Whole blood components vs blood component therapy** 33](#_Toc98756314)

**Criteria for Downgrading – GRADE Approach**

**risk of bias:**

**-1** high or unclear risk of selection (randomizzazione e allocazione) or outcome reporting bias

**-2** high or unclear risk of selection (randomizzazione e allocazione) and outcome reporting bias

**imprecision:**

**-1** events<200 or < 400 patients or confidence intervals crossed the line of no difference with plausible effects in favor to the experimental/group or wide confidence intervals

**-2** al least two of the above conditions

**indirectness:**

**-1** for setting (e.g., in-hospital)

**-2** for setting (e.g., in-hospital) and not enough information for PICO description (e.g., trauma and no-truama)

**inconsistency:**

**-1** for statistical inconsistency I^2^ >75% or methodological inconsistency (e.g., RCT and observational studies pooled together)

**-2** for statistical inconsistency I^2^ >90%

**publication bias:**

**-1** If n studies>10 and the Sr did not investigate the publication bias

**-2** If high risk of publication bias

**In case of observational studies, we upgrade for the following domains:**

**Large magnitude of effect:** This is a result of a study or meta-analysis and should not be used as a quality criterion. Large magnitude of effect may imply a high risk of biased results rather than increased confidence in results

**Dose-response gradient**: often exists in studies assessing etiology of disease, but effectiveness of an intervention usually does not show a linear dose-response pattern

**Residual confounding**: would further support inferences regarding treatment effect if some plausible confounders have not been documented, there is no credible way to determine how adjusting these parameters would alter the effectiveness estimates.

## Comparison 1: Hypertonic crystalloids vs isotonic crystalloids

|  |  | **Safiejko 2020** |
| --- | --- | --- |
| 1a | **Survival 24 h** | 4 RCTs, n=807, OR = 2.99; 95% CI: 2.04–4.39; i2 = 0%; p < 0.001 |
|  | risk of bias | Not serious |
|  | imprecision | Not serious |
|  | indirectness | Not serious |
|  | inconsistency | Not serious |
|  | publication bias | None |
|  | Other considerations |  |
|  | **QUALITY of EVIDENCE** | **HIGH** |
| 1b | **Survival 28 – 30 days** | 5 RCTs, n=1440, OR = 1.13; 95% CI: 0.75–1.70; i2 = 54%; p=0.56 |
|  | risk of bias | Not serious |
|  | imprecision | Serious |
|  | indirectness | Not serious |
|  | inconsistency | Not serious |
|  | publication bias | None |
|  | Other consideration |  |
|  | **QUALITY of EVIDENCE** | **MODERATE** |
| 1c | **Overall mortality** | 7 RCTs, n=1962, OR = 0.76; 95% CI: 0.61–0.94; i2=33%; p = 0.01 |
|  | risk of bias | Not serious |
|  | imprecision | Not serious |
|  | indirectness | Not serious |
|  | inconsistency | Not serious |
|  | publication bias | None |
|  | Other considerations |  |
|  | QUALITY of EVIDENCE | **HIGH** |

## Comparison 2a: Use of blood components

**2.1 Packed red blood cells (PRBCs) versus standard care**

|  |  | **Rijnhout 2019** |
| --- | --- | --- |
| 1a | **Mortality at 24 hours** | 3 retrospective studies, n=1029, OR = 0.92; 95% CI, 0.46–1.85; P = 0.82; i2=80% |
|  | risk of bias | Serious |
|  | imprecision | Serious |
|  | indirectness | Not serious |
|  | inconsistency | Serious |
|  | publication bias | None |
|  | Other consideration | None |
|  | **QUALITY of EVIDENCE** | **VERY LOW** |
| 1b | **Mortality at long term (30 days/1month and 12 months)** | 4 retrospective studies, n=1748, OR = 1.18; 95% CI, 0.93–1.49; P = 0.17; i2=18% |
|  | risk of bias | Serious |
|  | imprecision | Serious |
|  | indirectness | Not serious |
|  | inconsistency | Not serious |
|  | publication bias | None |
|  | Other consideration | None |
|  | **QUALITY of EVIDENCE** | **VERY LOW** |

**2.2 Packed red blood cells (PRBCs) + plasma versus standard care**

|  |  | **Rijnhout 2019** | |
| --- | --- | --- | --- |
| 1a | **Mortality at 24 hours** |  | 2 retrospective studies, n=495  OR = 0.47, 95% CI, 0.17–1.34; P = 0.16; i2=48% |
|  | risk of bias |  | Not serious |
|  | imprecision |  | Very serious |
|  | indirectness |  | Not serious |
|  | inconsistency |  | Not serious |
|  | publication bias |  | Not serious |
|  | Other consideration |  | None |
|  | **QUALITY of EVIDENCE** |  | **VERY LOW** |
| 1b | **Mortality at long term (30 days/1month and 12 months)** | 1 RCT, n=373  OR = 0.51; 95% CI, 0.33–0.81; P < 0.0001 | 3 retrospective studies, n=689  OR = 0.49; 95% CI, 0.29–0.83 P = 0.008; i2=0% |
|  | risk of bias | Not serious | Not serious |
|  | imprecision | Serious | Serious |
|  | indirectness | Not serious | Not serious |
|  | inconsistency | Not serious | Not serious |
|  | publication bias | Not serious | Not serious |
|  | Other consideration |  | None |
|  | **QUALITY of EVIDENCE** | **MODERATE** | **LOW** |

**2.3 Plasma versus standard care**

|  |  | **Rijnhout 2019** | **Coccolini 2019** |
| --- | --- | --- | --- |
| 1a | **Mortality at 24 hours** | 1 RCT, n=125  More patients in the plasma group died than in the control group, but not significantly (p=0.68) | 2 RCTs, n=626  RR = 0.69; 95% CI = 0.48–0.99; i2=34% |
|  | risk of bias | Not serious | Not serious |
|  | imprecision | Very serious | Serious |
|  | indirectness | Not serious | Not serious |
|  | inconsistency | Not serious | Not serious |
|  | publication bias | Not serious | None |
|  | **QUALITY of EVIDENCE** | **LOW** | **MODERATE** |
| 1b | **Mortality at 30 days/1month** | 1 RCT, n=125  More patients in the plasma group died than in the control group, but not significantly (p=0.37) | 2 RCTs, n=626  RR = 0.86; 95% CI = 0.68–1.11; i2=38% |
|  | risk of bias | Not serious | Not serious |
|  | imprecision | Very serious | Very serious |
|  | indirectness | Not serious | Not serious |
|  | inconsistency | Not serious | Not serious |
|  | publication bias | Not serious | Not serious |
|  | **QUALITY of EVIDENCE** | **LOW** | **LOW** |

## Comparison 2b: Blood components ratios

**2.4 FFP:PLT:PRBC ratio**

|  |  | **McQuilten 2018** | **Luz 2019** | **Ritchie 2020** |
| --- | --- | --- | --- | --- |
| 1a | **Mortality 24 h** | 1 RCTs, n=680; RR = 0.75; 95% CI: 0.52-1.08 | 1 RCTs, n=680;  difference: -5.4% (95% CI -10.4% to -0.5%; p = 0.03 | 1 RCT, n=680;  RR = 1.33; 95% CI: 0.93–1.92 |
|  | risk of bias | Serious | Serious | Serious |
|  | imprecision | Very serious | Serious | Very serious |
|  | indirectness | Not serious | Not serious | Not serious |
|  | inconsistency | Not serious | Not serious | Not serious |
|  | publication bias | None | None | None |
|  | **QUALITY of EVIDENCE** | **VERY LOW** | **LOW** | **VERY LOW** |
| 1b | **Mortality at 1 month** | 2 RCTs, n=755; RR = 1.26; 95% CI: 0.49-3.22, i2=75% | 2 RCTs, n=755;  OR = 1.35; 95% CI: 0.40-4.59, i2=76% | 1 RCT, n=680;  RR = 1.17; 95% CI: 0.90–1.53 |
|  | risk of bias | Serious | Not serious | Serious |
|  | imprecision | Very serious | Very serious | Very serious |
|  | indirectness | Not serious | Not serious | Not serious |
|  | inconsistency | Serious | Serious | Serious |
|  | publication bias | None | None | None |
|  | **QUALITY of EVIDENCE** | **VERY LOW** | **VERY LOW** | **VERY LOW** |

**2.5 FFP:PRBC ratio, 1:1 vs <1:1**

|  |  | **Luz 2019** | **Rahouma 2018*** |
| --- | --- | --- | --- |
| 1a | **Mortality at 24 hours** | 5 observational studies, n=2414  OR = 0.34; 95% CI: 0.14-0.82; i2=88% | 1 RCT and 6 observational studies, n=5265  OR = 2.05; 95% CI: 1.55-2.71, i2=57% |
|  | risk of bias | Not serious | Not serious |
|  | imprecision | Not serious | Not serious |
|  | indirectness | Not serious | Not serious |
|  | inconsistency | Serious | Not serious |
|  | publication bias | None | None |
|  | **QUALITY of EVIDENCE** | **VERY LOW** | **LOW** |
| 1b | **Mortality at 1 month** | 10 observational studies, n=4203  OR = 0.38; 95% CI: 0.22-0.68); i2=91% | 2 RCTs and 5 observational studies, n=5266  OR = 1.36; 95% CI: 1.09-1.69); i2=45% |
|  | risk of bias | Not serious | Not serious |
|  | imprecision | Not serious | Not serious |
|  | indirectness | Not serious | Not serious |
|  | inconsistency | Serious | Not serious |
|  | publication bias | None | None |
|  | **QUALITY of EVIDENCE** | **VERY LOW** | **LOW** |

* **<1:1 vs 1:1**

**2.6 FFP:PRBC ratio, 1:1.5 vs <1:1.5**

|  |  | **Luz 2019** | **Rahouma 2018*** |
| --- | --- | --- | --- |
| 1a | **Mortality at 24 hours** | 2 observational studies, n=118  OR = 0.43; 95% CI: 0.18-1.06; i2=0% | 4 observational studies, n=1877  OR = 3.97; 95% CI: 1.37-11.49; i2=88% |
|  | risk of bias | Not serious | Not serious |
|  | imprecision | Very serious | Not serious |
|  | indirectness | Not serious | Not serious |
|  | inconsistency | Not serious | Serious |
|  | publication bias | None | None |
|  | **QUALITY of EVIDENCE** | **VERY LOW** | **VERY LOW** |
| 1b | **Mortality at 1 month** | 5 observational studies, n=1369  OR = 0.42; 95% CI: 0.22-0.81); i2=73% | 5 observational studies, n=1813  OR = 2.45; 95% CI: 1.14-5.25); i2=87% |
|  | risk of bias | Not serious | Not serious |
|  | imprecision | Not serious | Not serious |
|  | indirectness | Not serious | Not serious |
|  | inconsistency | Serious | Serious |
|  | publication bias | None | None |
|  | **QUALITY of EVIDENCE** | **VERY LOW** | **VERY LOW** |

* **<1:1.5 vs 1:1.5**

**2.7 FFP:PRBC ratio, 1:2 vs <1:2**

|  |  | **Luz 2019** | **Rahouma 2018*** |
| --- | --- | --- | --- |
| 1a | **Mortality at 24 hours** | 6 observational studies, n=1388  OR = 0.59; 95% CI: 0.43-0.81; i2=22% | 9 observational studies, n=3540  OR = 2.85; 95% CI: 2.14-3.81; i2=59% |
|  | risk of bias | Not serious | Not serious |
|  | imprecision | Not serious | Not serious |
|  | indirectness | Not serious | Not serious |
|  | inconsistency | Not serious | Not serious |
|  | publication bias | None | None |
|  | **QUALITY of EVIDENCE** | **LOW** | **LOW** |
| 1b | **Mortality at 1 month** | 10 observational studies, n=2849  OR = 0.47; 95% CI: 0.31-0.71); i2=81% | 14 observational studies, n=6193  OR = 1.77; 95% CI: 1.50-2.10; i2=37% |
|  | risk of bias | Not serious | Not serious |
|  | imprecision | Not serious | Not serious |
|  | indirectness | Not serious | Not serious |
|  | inconsistency | Serious | Not serious |
|  | publication bias | None | None |
|  | **QUALITY of EVIDENCE** | **VERY LOW** | **LOW** |

* **<1:2 vs 1:2**

**2.8 PLT:PRBC ratio, 3.3.1 ≥1:1 vs < 1:1**

|  |  | **Luz 2019** |
| --- | --- | --- |
| 1a | **Survival and free massive transfusion 24 h** | 1 observational study, n=385  OR = 2.67; 95% CI: 1.24-5.77; |
|  | risk of bias | Not serious |
|  | imprecision | Serious |
|  | indirectness | Not serious |
|  | inconsistency | Not serious |
|  | publication bias | None |
|  | **QUALITY of EVIDENCE** | **VERY LOW** |

**2.9 PLT:PRBC ratio, ≥1:9 vs < 1:9**

|  |  | **Luz 2019** |
| --- | --- | --- |
| 1a | **Mortality 24 h** | 1 observational study, n=604  administration within 6 h: HR = 0.34; 95% CI: 0.11–0.89  administration within 12 h: HR = 0.17; 95% CI: 0.04–0.54  administration within 24 h: HR = 0.26; 95% CI: 0.06–0.86 |
|  | risk of bias | Not serious |
|  | imprecision | Serious |
|  | indirectness | Not serious |
|  | inconsistency | Not serious |
|  | publication bias | None |
|  | **QUALITY of EVIDENCE** | **VERY LOW** |

**2.10 PLT:PRBC ratio, >1:20 vs 1:1**

|  |  | **Luz 2019** |
| --- | --- | --- |
| 1a | **Mortality 24 h** | 1 observational study, n=643  RR = 2.81; 95% CI: 1.36–5.8 |
|  | risk of bias | Not serious |
|  | imprecision | Serious |
|  | indirectness | Not serious |
|  | inconsistency | Not serious |
|  | publication bias | None |
|  | **QUALITY of EVIDENCE** | **VERY LOW** |
| 1b | **Mortality 30 days** | 1 observational study, n=643  RR = 1.77; 95% CI: 1.16–2.68 |
|  | risk of bias | Not serious |
|  | imprecision | Serious |
|  | indirectness | Not serious |
|  | inconsistency | Not serious |
|  | publication bias | None |
|  | **QUALITY of EVIDENCE** | **VERY LOW** |

**2.11 PLT:PRBC ratio, 1:2 vs 1:1**

|  |  | **Luz 2019** |
| --- | --- | --- |
| 1a | **Mortality 24 h** | 1 observational study, n=643  RR = 3.13; 95% CI: 1.52–6.45 |
|  | risk of bias | Not serious |
|  | imprecision | Serious |
|  | indirectness | Not serious |
|  | inconsistency | Not serious |
|  | publication bias | None |
|  | **QUALITY of EVIDENCE** | **VERY LOW** |
| 1b | **Mortality 30 days** | 1 observational study, n=643  RR = 1.75; 95% CI: 1.15–2.65 |
|  | risk of bias | Not serious |
|  | imprecision | Not serious |
|  | indirectness | Not serious |
|  | inconsistency | Not serious |
|  | publication bias | None |
|  | **QUALITY of EVIDENCE** | **LOW** |

**2.12 PLT:PRBC ratio, 1:8 vs 1:16**

|  |  | **Luz 2019** |
| --- | --- | --- |
| 1a | **Mortality 24 h** | 1 observational study, n=462  RR = 0.82; 95% CI: 0.72–0.93 |
|  | risk of bias | Not serious |
|  | imprecision | Serious |
|  | indirectness | Not serious |
|  | inconsistency | Not serious |
|  | publication bias | None |
|  | **QUALITY of EVIDENCE** | **VERY LOW** |
| 1b | **Mortality 30 days** | 1 observational study, n=462  RR = 0.91; 95% CI: 0.86–0.95 |
|  | risk of bias | Not serious |
|  | imprecision | Serious |
|  | indirectness | Not serious |
|  | inconsistency | Not serious |
|  | publication bias | None |
|  | **QUALITY of EVIDENCE** | **VERY LOW** |

**2.13 PLT:PRBC ratio, ≥ 1:2 vs <1:2**

|  |  | **Luz 2019** |
| --- | --- | --- |
| 1a | **Survival 30 days** | 1 observational study, n=214  OR = 1.55; 95% CI: 1.09–2.18 |
|  | risk of bias | Not serious |
|  | imprecision | Serious |
|  | indirectness | Not serious |
|  | inconsistency | Not serious |
|  | publication bias | None |
|  | **QUALITY of EVIDENCE** | **VERY LOW** |
| 1b | **Mortality 30 days** | 1 observational study, n=526  HR = 0.63; 95% CI: 0.39–0.86 |
|  | risk of bias | Not serious |
|  | imprecision | Serious |
|  | indirectness | Not serious |
|  | inconsistency | Not serious |
|  | publication bias | None |
|  | **QUALITY of EVIDENCE** | **VERY LOW** |

**2.14 PLT:PRBC ratio, ≥0.1 vs ≤0.1**

|  |  | **Wirtz 2019** |
| --- | --- | --- |
| 1a | **Survival 24 h** | 1 observational study, n=414  HR = 4.25; 95% CI: 1.25–14.48 |
|  | risk of bias | Serious |
|  | imprecision | Serious |
|  | indirectness | Not serious |
|  | inconsistency | Not serious |
|  | publication bias | None |
|  | **QUALITY of EVIDENCE** | **VERY LOW** |
| 1b | **Survival 30 days** | 1 observational study, n=414  HR = 2.32; 95% CI: 1.11–4.48 |
|  | risk of bias | Serious |
|  | imprecision | Serious |
|  | indirectness | Not serious |
|  | inconsistency | Not serious |
|  | publication bias | None |
|  | **QUALITY of EVIDENCE** | **VERY LOW** |

**2.15 PLT:PRBC ratio, ≥0.3 vs <0.3**

|  |  | **Rijnhout 2021** |
| --- | --- | --- |
| 1a | **Mortality at 24 hours** | 2 observational studies, n=413  OR = 0.12; 95% CI: 0.08–0.21; p < 0.00001; i2=0% |
|  | risk of bias | Serious |
|  | imprecision | Serious |
|  | indirectness | Not serious |
|  | inconsistency | Not serious |
|  | publication bias | None |
|  | **QUALITY of EVIDENCE** | **VERY LOW** |

**2.16 PLT:PRBC ratio, ≥0.5 vs <0.5**

|  |  | **Rijnhout 2021** |
| --- | --- | --- |
| 1a | **Mortality at 24 hours** | 4 observational studies, n=2143  OR = 0.46; 95% CI: 0.28–0.76; p=0.002; i2=75% |
|  | risk of bias | Serious |
|  | imprecision | Not serious |
|  | indirectness | Not serious |
|  | inconsistency | Serious |
|  | publication bias | None |
|  | **QUALITY of EVIDENCE** | **VERY LOW** |
| 1b | **Mortality at 30 days** | 2 observational study, n=1117  OR = 0.68; 95% CI: 0.50–0.91; p=0.01; i2=0% |
|  | risk of bias | Serious |
|  | imprecision | Not serious |
|  | indirectness | Not serious |
|  | inconsistency | Not serious |
|  | publication bias | None |
|  | **QUALITY of EVIDENCE** | **VERY LOW** |

**2.17 PLT:PRBC ratio, ≥1 vs 0.5 or < 1**

|  | SR | **Rijnhout 2021** |
| --- | --- | --- |
| 1a | **Mortality at 24 hours** | 2 RCTs and 1 observational study, n=1497  OR = 0.81; 95% CI: 0.30–2.19; p=0.68; i2=93% |
|  | risk of bias | Serious |
|  | imprecision | Serious |
|  | indirectness | Not serious |
|  | inconsistency | Serious |
|  | publication bias | None |
|  | **QUALITY of EVIDENCE** | **VERY LOW** |

**2.18 PLT:PRBC ratio, ≥1 vs 0.6 or < 1**

|  |  | **Rijnhout 2021** |
| --- | --- | --- |
| 1a | **Mortality at 1 month** | 2 RCTs and 1 observational study, n=1181  OR = 0.58; 95% CI: 0.35–0.98; p=0.04; i2=64% |
|  | risk of bias | Serious |
|  | imprecision | Not serious |
|  | indirectness | Not serious |
|  | inconsistency | Not serious |
|  | publication bias | None |
|  | **QUALITY of EVIDENCE** | **VERY LOW** |

**2.19 PLT:PRBC ratio, HIGH vs LOW**

|  | SR | **Kleinveld 2021** |
| --- | --- | --- |
| 1a | **Mortality at 24 hours** | 5 RCTs, n=1757  OR = 0.69; 95% CI: 0.53–0.89; i2=41% |
|  | risk of bias | Serious |
|  | imprecision | Not serious |
|  | indirectness | Not serious |
|  | inconsistency | Not serious |
|  | publication bias | None |
|  | **QUALITY of EVIDENCE** | **MODERATE** |
| 1a | **Mortality at 1 month** | 5 RCTs, n=1757  OR = 0.78; 95% CI: 0.63–0.98; i2=47% |
|  | risk of bias | Serious |
|  | imprecision | Not serious |
|  | indirectness | Not serious |
|  | inconsistency | Not serious |
|  | publication bias | None |
|  | **QUALITY of EVIDENCE** | **MODERATE** |

## Comparison 3: Whole blood components vs blood component therapy

|  |  | **McQuilten 2020** | **Crowe 2020** | **Cruciani 2020** |
| --- | --- | --- | --- | --- |
| 1a | **Mortality at 24 hours** | 1 RCT, n=107  RR = 1.13; 95% CI: 0.37-3.49 | 1 RCT and 4 observational studies  OR = 0.83; 95% CI: 0.56–1.24; i2=27.2%. | 1 RCT and 2 observational studies, n=782  OR = 0.80; 95% CI: 0.40-1.59; p=0.53; i2 = 31% |
|  | risk of bias | Very serious | Very serious | Very serious |
|  | imprecision | Very serious | Serious | Very serious |
|  | indirectness | Not serious | Not serious | Not serious |
|  | inconsistency | Not serious | Serious | Serious |
|  | publication bias | None | None | None |
|  | Other consideration |  | No | No |
|  | **QUALITY of EVIDENCE** | **VERY LOW** | **VERY LOW** | **VERY LOW** |
| 1b | **Mortality 30 days** | 1 RCT, n=107  RR = 1.42; 95% CI: 0.63-3.19 | 1 RCT and 11 observational studies  OR = 0.79; 95% CI: 0.49–1.31; i2 = 87.3% | 1 RCT and 6 observational studies, n=3642  OR = 0.90; 95% CI: 0.62-1.30; p=0.56: i2 = 56% |
|  | risk of bias | Very serious | Very serious | Very serious |
|  | imprecision | Very serious | Serious | Serious |
|  | indirectness | Not serious | Not serious | Not serious |
|  | inconsistency | Not serious | Serious | Serious |
|  | publication bias | None | None | None |
|  | Other consideration |  | No | No |
|  | **QUALITY of EVIDENCE** | **VERY LOW** | **VERY LOW** | **VERY LOW** |
